# Elucidating the role of pyrabactin-like receptors of finger millet under drought and salinity stress: an insight into *in silico*, machine learning and molecular approaches

**DOI:** 10.3389/fgene.2025.1598523

**Published:** 2025-05-29

**Authors:** Varsha Rani, Theivanayagam Maharajan, Shefali Singh, D. C. Joshi, Ramwant Gupta, Dinesh Yadav

**Affiliations:** ^1^ Department of Biotechnology, Deen Dayal Upadhyaya Gorakhpur University, Gorakhpur, Uttar Pradesh, India; ^2^ Department of Biotechnology, School of Engineering and Technology, Sandip University, Nashik, Maharashtra, India; ^3^ Division of Plant Molecular Biology and Biotechnology, Department of Biosciences, Rajagiri College of Social Sciences, Kochi, Kerala, India; ^4^ ICAR-Vivekananda Institute of Hill Agriculture, Almora, Uttarakhand, India; ^5^ Department of Botany, Deen Dayal Upadhyaya Gorakhpur University, Gorakhpur, Uttar Pradesh, India

**Keywords:** ABA receptor, ABA signaling pathways, abiotic stress, finger millet, PYL gene family

## Abstract

**Introduction:**

The Pyrabactin Resistance 1-like (PYL) receptors, a family of proteins in plants, play a vital role in abscisic acid (ABA) signalling, assisting plants in managing abiotic stresses. Finger millet (*Eleusine coracana* (L.) Gaertn) is a naturally drought tolerant crop, yet the receptor proteins involved in its stress signalling pathways remain poorly understood.

**Method:**

This study employed bioinformatics, machine learning, and molecular approaches to identify, characterize, and profile the expression of PYL receptors in response to drought and salinity stress.

**Results:**

The study identified 14 *PYL* genes in the finger millet genome, irregularly distributed across four of the nine mapped chromosomes. Phylogenetic analysis grouped these genes into three subfamilies. Machine learning analysis highlighted five putative PYL genes—*EcPYL4-2A, EcPYL7-2B, EcPYL11-5A, EcPYL12-5A*, and *EcPYL14-5B* with expression levels exceeding 70% under drought and salinity stress. These genes were further validated through qRT-PCR, confirming their expression under stress conditions, though expression levels varied across tissues and genes.

**Discussion:**

The identification of PYL genes responsive to drought and salt stress provides valuable insights into the stress-signalling mechanisms of finger millet. Among the identified genes, *EcPYL7-2B* and *EcPYL12-5A* emerged as promising candidates for further characterization through genome editing and molecular approaches. This study highlights the potential of these genes in enhancing the stress resilience of finger millet, contributing to its role in improving food and nutritional security under challenging environmental conditions.

## Introduction

Finger millet (*Eleusine coracana* L. Gaertn.), an ancient grain, has considerable historical, cultural, and nutritional significance, especially in Asia and Africa (Sood et al., 2019). Farmers widely cultivate it in arid and semi-arid regions for their daily diet and to fulfill its nutritional properties for their daily life (Goron and Raizada, 2015). Finger millet is widely recognized for its capacity to thrive in resource-scarce environments and its exceptional nutritional value. Amidst climate change and population expansion, the stability of finger millet production is essential for ensuring global food security and socio-economic development (Rani et al., 2023; Sanjay Kumar et al., 2024).

The growth and yield of finger millet are primarily influenced by biotic and abiotic stresses (Rani et al., 2023). As finger millet has evolved over time, it has developed complex signal networks which regulate a number of biochemical and physiological processes imparting tolerance to biotic and abiotic stresses (Xiong et al., 2002; Hittalmani et al., 2017). Studying the expression pattern and role of key candidate genes associated with stress tolerance will be helpful in developing new stress responsive finger millet genotypes.

Abscisic acid (ABA) is a phytohormone that has a huge impact on plant development and growth. It is an important hormone that controls organ senescence and morphogenesis, stomatal movement, and transpiration (Cutler et al., 2010; Zhu, 2016). In addition to acting as a stress indicator for plants, ABA may also be used to identify drought, salinity, or pathogen infection (Zhu, 2002; Cutler et al., 2010). In response to abiotic stress, the accumulation of ABA increases in plants. Generally, plants perceive ABA through the Pyrabactin Resistance 1 (PYR1)/PYR1-like (PYL)/Regulatory component of the ABA receptor (RCAR) protein family. After binding to PYL, ABA changes the conformation of PYL, which facilitates its binding to type 2C protein phosphatases (PP2Cs). This allows SnRK2s to be released from PP2C inhibition. Yadav et al. (2020) reported that ABA stimulates downstream targets like transcription factors (TF) and other regulatory proteins because it turns on SnRK2. The stimulated TF then enters the nucleus, up-regulating downstream genes associated with ABA-induced stress. Hence, land plants require PYLs, PP2Cs, and SnRK2s for ABA signalling to survive under abiotic stresses like drought, salinity, and others (Cutler et al., 2010).

PYLs have been identified and characterized in *Arabidopsis* (Gonzalez-Guzman et al., 2012), rice (Kim et al., 2012; Yadav et al., 2020), sorghum (Dalal and Inupakutika, 2014), maize (Fan et al., 2018; Wang et al., 2018) and wheat (Lei et al., 2021). Some plants, like *Arabidopsis*, have a gene called AtPYR1 that codes for a cyclase subfamily member and is part of the star related lipid transfer (START) domain superfamily (Park et al., 2009). *AtPYL* (PYR1-like) genes *AtPYL1* to *AtPYL13* are genes that share the same function as *AtPYR*1. In *Arabidopsis* PYL family, there are 14 members, each having a START domain. These 14 PYLs are divided into three subfamilies (Park et al., 2009; Park et al., 2010). It has been observed that some *AtPYLs* form monomers which interact with phosphatase 2C (Bai et al., 2013) protein without the involvement of ABA. In addition, some *AtPYLs* can form homodimers that only interact with PP2C after binding to ABA (Hao et al., 2011). It has been reported that overexpression of AtPYL4 enhanced drought tolerance in *Arabidopsis*. More interestingly, overexpression of OsPYL5 in rice improved drought and salinity tolerance (Pizzio et al., 2013; Kim et al., 2014). These results suggest that the PYL family plays an important role in plant development and abiotic stress response. However, the role of PYL receptors in finger millet under abiotic stresses is still unknown, and needs to be deciphered. Identification and characterization of *PYL* genes across the finger millet genome can provide an insight into the response of finger millet towards stresses and role of ABA signaling pathways.

Several studies have reported that finger millet is drought-tolerant, yet further research is required to identify how genes and regulatory proteins contribute to stress impartation in finger millet (Mukami et al., 2019; Talwar et al., 2020; Rani et al., 2024a; Rani et al., 2024c; Rani et al., 2024b). The draft and annotated genome sequence of finger millet has been made public and openly accessible (Hittalmani et al., 2017; Hatakeyama et al., 2018); however, many genes are still unannotated and need to be studied using *in silico* and wet lab methods. To the best of our knowledge, the role of PYL receptors in finger millet, has not been reported. The ultimate aim of the study is to reveal the role of PYL receptors in finger millet through *in silico*, machine learning and molecular approaches.

The advancement of bioinformatics and computational tools has enabled the integration of machine learning (ML) into stress biology, offering new opportunities for predicting gene expression patterns under adverse environmental conditions. This study provides an insight into the role of Pyrabactin-Like Receptors (PYL) in finger millet under drought and salinity stress with involvement of ABA signaling pathways using a comprehensive approach comprising of *in silico*, machine learning, and molecular tools. The finger millet genome was mined for several bioinformatics attributes like phylogenetic analysis, chromosomal localization, gene structure mapping, motif analysis, cis-regulatory element identification, protein-protein interaction analysis, miRNA target predictions and digital expression profiling using machine learning algorithm of ASRpro web server (Meher et al., 2024). Further, the most promising PYL candidate genes, likely to be expressed under drought and salinity stress, has been identified revealing the possible plant stress adaptation mechanisms. Furthermore, qRT-PCR validation was conducted for the identified candidate genes in shoots and roots of finger millet exposed to drought and salinity stress. The identification of key PYL receptors in finger millet could reveal the possible molecular mechanisms underlying its resilience to abiotic stresses.

## Materials and methods

### Collection and characterization of *PYL* genes of finger millet

Firstly, we obtained the PYL full-length protein sequence of rice from the NCBI (http://www.ncbi.nlm.nih.gov/nucleotide/) (Yadav et al., 2020). The rice *PYL*s gene sequences were used as query sequences in the Phytozome database (https://phytozome-next.jgi.doe.gov/) to find PYLs in the finger millet genome. This was done by using BLASTp searches (http://blast.ncbi.nlm.nih.gov/) against the finger millet genome *E. coracana* v1.1 (Goodstein et al., 2012; Hittalmani et al., 2017; Hatakeyama et al., 2018). The appeared duplicate and redundant sequences were eliminated by venny2.1 (https://bioinfogp.cnb.csic.es/tools/venny/) (Oliveros, 2016). Then, the NCBI open reading frames (ORF) Finder (http://www.ncbi.nlm.nih.gov/gorf/gorf.html) was used to determine ORFs. On the other hand, we used Pfam (https://www.ebi.ac.uk/interpro/entry/pfam/) to search for the polyketide cyclase 2/START domain (PF10604), which is a characteristic of the PYR/PYL protein sequence. Further, multiple sequence alignment by MEGA11 was used to identify conserved amino acid residues among all putative PYL sequences of finger millet (Tamura et al., 2021). Genes that lack specific conserved domains were sorted and removed. Further the domain position, physicochemical attributes and subcellular localization were predicted by HMMSCAN (http://www.ebi.ac.uk/Tools/hmmer/search/hmmscan, ExPaSy Protparam (https://web.expasy.org/protparam/) and Wolf-Psort online tool (https://www.genscript.com/tools/wolf-psort) respectively (Gasteiger et al., 2005; Horton et al., 2007).

### Evolutionary analysis, gene structure display and motif prediction of *PYL* genes

The *PYL* gene family in finger millet has been designated as EcPYL1-1A, which indicates for the first *PYL* genes of *E. coracana* and is found on chromosome 1A. Similarly, other finding was also annotated and used for evolutionary study. An evolutionary analysis was made between putative *PYLs* genes of finger millet and the *PYLs* genes of rice (Yadav et al., 2020), sorghum (Dalal and Inupakutika, 2014), and foxtail millet (Duarte et al., 2019), which were retrieved from the Phyozome and NCBI database. All of the retrieved sequences of the *PYL* genes family were aligned through ClustalW, and the phylogenetic tree was constructed through MEGA11 using the Maximum Likelihood method at 1,000 bootstrap levels (Larkin et al., 2007; Tamura et al., 2021). MapGene2 Chromosome v2.1 (http://mg2c.iask.in/mg2c_v2.1/) was used for physical mapping of *PYLs* gene of finger millet (Devos et al., 2023), and TB tool was used to display the exon-intron structure (Chen et al., 2020; Chao et al., 2021). The MEME Suite Vs. 5.5.3 (https://meme-suite.org/meme/tools/meme) predicted the conserved motif within each finger millet protein sequence. The maximum number of motifs was set to 6, keeping others at default parameters (Bailey et al., 2015).

### Ka/Ks and gene duplication analysis of *PYL*s gene family of finger millet

A ratio of non-synonymous (Ka) versus synonymous (Ks) substitution rates of nucleotides was calculated among finger millet *PYLs* gene family by using Ka/Ks calculation tool (http://services.cbu.uib.no/tools/kaks) and the gene duplication event was calculated by using T = Ks/2λ), where (λ)= (6.5 × 10^−9^ (Gaut et al., 1996; Zhang et al., 2006). It measures the selective pressures placed on genes and can be used to identify pairs of genes where encoded proteins may have changed their function. When the ratio of Ka/Ks > 1, it indicates that there is a positive selective pressure or Darwinian selection. If the ratio is 1, it indicates neutral evolution and if the ratio is less than 1, then it indicates stabilizing selection, i.e., the protein sequence is pressured to be conserved.

### Prediction of cis-regulatory elements in the promoters of finger millet

The 2,000 bp upstream sequences from the translations tart site of all of the *EcPYLs* genes were obtained from finger millet genome at Phytozome database (Goodstein et al., 2012) and the putative cis regulatory elements were predicted by using the PlantCARE web server (http://bioinformatics.psb.ugent.be/webtools/plantcare/html/) to know the putative function associated with the genes (Lescot et al., 2002). According to the cis-regulatory elements, *PYL* genes were categorized into three groups based on their response to phytohormones, stress, and growth and development.

### miRNA-PYLs target prediction and protein-protein interaction and gene ontology analysis

For the prediction of miRNA target sites in the *EcPYL* genes, the miRNA dataset of finger millet was retrieved from PmiREN server (https://www.pmiren.com/) (Guo et al., 2020) and cDNA sequence of *EcPYLs* genes were examined against miRNA dataset using the pSRNATarget server (https://www.zhaolab.org/psRNATarget/) (Dai and Zhao, 2011). To determine the interrelationships among the 14 putative *EcPYLs* genes, the STRING database (https://string-db.org/) (Szklarczyk et al., 2021) was used at confidence level (0.07) against rice (Yu et al., 2002), *Arabidopsis* (Cheng et al., 2017), sorghum (McCormick et al., 2018) and foxtail millet (Zhang et al., 2012) genome one by one. Using the combination of Cytoscape v. 3.10.3 with MCODE (Molecular Complex Detection), the most accurate prediction of the interaction network was displayed. Additionally, the integration of Cytoscape v. 3.10.3 with BinGO program were used for Gene Ontology (GO), functional annotation and gene enrichment profiling of *EcPYL* genes of finger millet (Paul et al., 1971; Bader and Hogue, 2003).

### Machine learning approach for preselection of candidate genes

After characterization of *EcPYL* genes, machine learning algorithm was used to predict the expression pattern of these genes. For the prediction of probability of expression of *EcPYL* genes under drought and salinity stresses we used ASRPro webserver (http://iasri-sg.icar.gov.in/asrpro/result.php) (Meher et al., 2024). This web server utilized machine learning algorithms to assess the likelihood of gene expression under the given stress conditions, and generated a probability score for each gene. Genes with a probability score of ≥0.70 were considered highly likely to be expressed in response to drought and salinity stress. On the basis of the probability of gene expression, we constructed a heatmap and scatter plot to predict the candidate genes for qRT-PCR expression profiling.

### Gene expression analysis

Five *PYL* genes (*EcPYL4-2A, EcPYL7-2B, EcYL11-5A, EcPYL12-5A,* and *EcPYL14-5B*) with probability score of 0.70 were selected for qRT-PCR analysis. Primers for each gene were designed using Primer3 input software (https://bioinfo.ut.ee/primer3-0.4.0/), and the primers specificity was done with genome sequences of finger millet to ensure that primers only bind to the intended target sequence. The primer sequences, annealing temperature, and product size of each primer were listed in Table 1. Healthy and viable seeds of finger millet genotype (IC87469) was selected for this experiment, and grown under drought (15% PEG-induced drought stress) and salinity (200 mM NaCl) stress conditions according to previous protocols (Rakkammal et al., 2023b; Rakkammal et al., 2023a). Both shoot and root tissues were harvested after 28 days of growth, and they were used for gene expression analysis. Total RNA was isolated from shoot and root tissues using the Trizol method (Rakkammal et al., 2023a; Rakkammal et al., 2023b), and cDNA was synthesized using the QuantiTect Reverse transcription kit (Qiagen, Germany). *EF-1α* was used as a reference gene to normalize the expression pattern of each *PYL* gene. Cycle threshold values of each *PYL* genes were analyzed using the formula 2^–∆∆Ct^. A total of three biological and three technical replicates were used for each treatment. Whereas, statistical analysis were performed through origin 2021 software (https://www.originlab.com/origin) using ANOVA, followed by Tukey test for multiple comparisons for significantly difference genes (*p* ≤ 0.05).

**TABLE 1:** Details of primers used for qRT-PCR analysis.

S. no.	Gene name	Forward primer	Reverse primer	Product size with intron	Product size without intron	Annealing temperature (°C)
1	*EcPYL4-2A*	ACC​TCG​TTT​GGT​CTC​TGG​TG	TCG​ATG​ATC​ACC​TCC​AAC​AA	-	215	60.0
2	*EcPYL7-2B*	CCT​GCT​ACA​CGA​AGC​ACT​GA	CGA​CTT​GAG​GTT​GCA​CTT​GA	1,284	249	60.1
3	*EcPYL11-5A*	GGA​TCA​CCA​CCA​TCA​CGA​G	CTC​CGG​ATG​AAG​TGC​TTG​TA	-	214	59.2
4	*EcPYL12-5A*	ACC​GCC​TCC​AGA​ACT​ACT​CA	CCT​CGA​CGA​AGT​AGC​AGG​TC	-	141	59.9
5	*EcPYL14-5B*	CAC​CGC​CTC​CAG​AAC​TAC​TC	CTG​CAA​GAG​ACG​TGA​GGT​TG	-	175	59.7

### Homology modeling of finger millet *PYLs* genes

The SWISS MODEL predicted the *in silico* structure of EcPYLs proteins (Waterhouse et al., 2018). PDBsum (http://www.ebi.ac.uk/thornton-srv/databases/pdbsum/Generate.html) and the SAVES v6.0 server (https://saves.mbi.ucla.edu/) were used to validate, analyze, and estimate the quality of the build model in two dimensions (Laskowski et al., 2018). Whereas, ResProx (http://www.resprox.ca/) (Berjanskii et al., 2012) was used to estimate the resolution of the model and SuperPose v. 1 (http://superpose.wishartlab.com/) and Biovia Discovery Studio 2019 was used for superposition and visualization of model, respectively.

## Results

### Sequence analysis of *PYL* genes in finger millet

**FIGURE 1:**
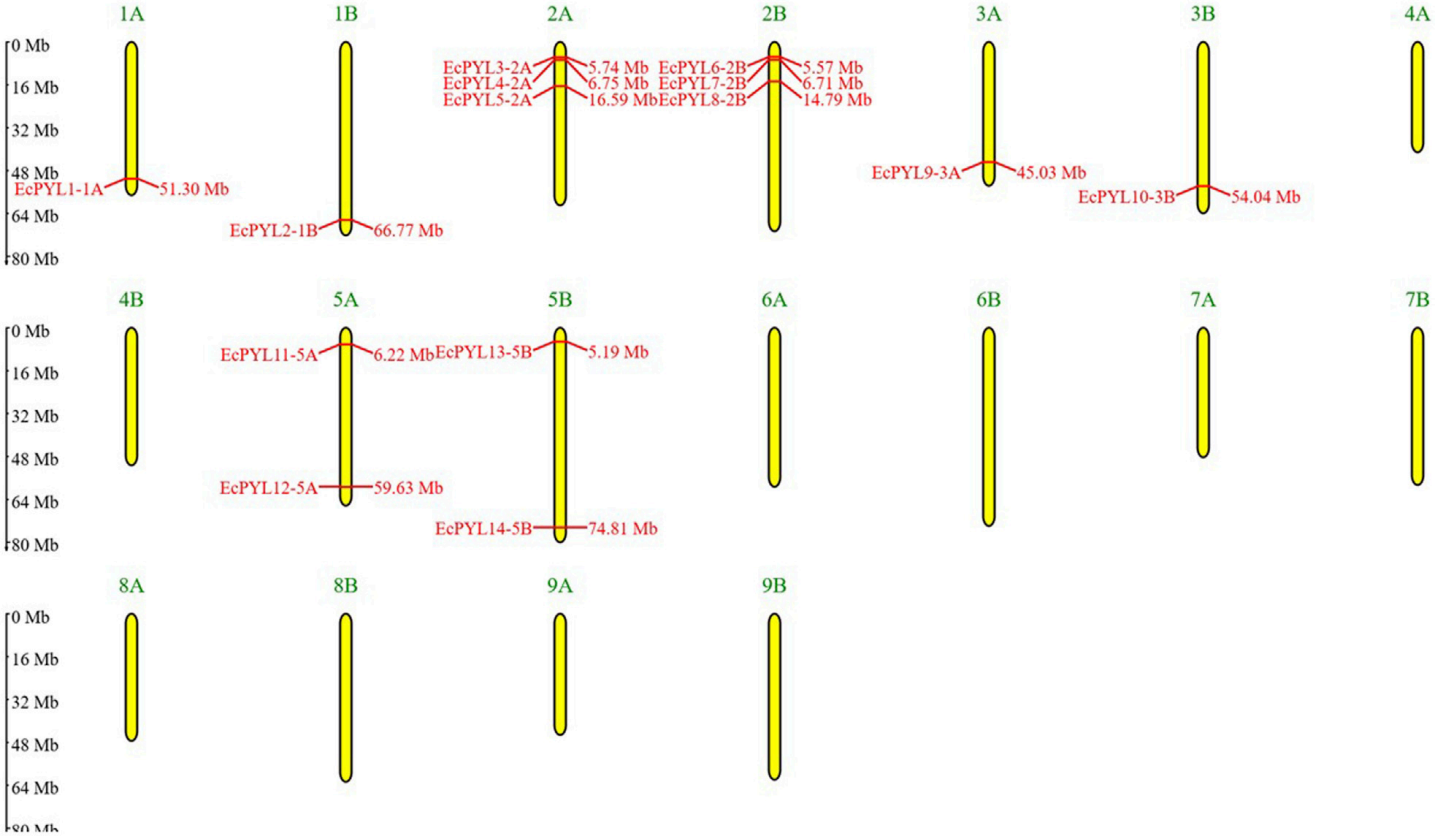
Distributions of 14 *EcPYL* genes on finger millet chromosomes. Physical map of putative *EcPYL* genes across nine chromosome groups found on the finger millet genome, each with a homologous chromosome **(A,B)**. All finger millet chromosomes are shown to scale based on their actual length.

A total of 27 *PYL* genes were retrieved from the finger millet genome (Supplementary Table S1). Moreover, based on conserved domain analysis, the redundant sequences were removed, and 14 *PYL* genes were considered for further *in silico* studies. Table 2 shows the gene position, ORF length, domain position, and chromosome location of these 14 *PYL* genes. These genes have the polyketide cyclase 2/START domain and have the Pfam accession number PF10604. These 14 putative *EcPYLs* genes were unevenly distributed among four out of nine sets of chromosomes (Figure 1). The chromosomes 2A and 2B possess three *EcPYL* genes, chromosomes 5A and 5B has two *EcPYL* genes while chromosomes 1A and 1B and 3A and 3B possess only one *EcPYL* gene. *EcPYL* genes were not observed on chromosomes 4, 6, 7, 8, and 9. In general, both A and B sub-genomes have seven *EcPYL* genes, showing that there is not much difference in the number of *PYL* genes at the sub-genome level. For a better understanding, we named the possible genes *EcPYL* (*E. coracana PYL*) followed by the number of chromosomes located, for example, *EcPYL_1A*. We identified 14 *EcPYL* genes in finger millet and sequentially named them as *EcPYL1 to EcPYL14*, followed by their respective chromosomal numbers. The physicochemical attributes of putative *PYLs* of finger millet (Table 3) revealed that *EcPYLs* ranged from 181 to 222 aa. Accordingly, the molecular weight ranged from 19.5 to 23.7 kDa, and PI ranged from 5.5 to 9.3. The ORF lengths predicted by the ORF finder for *EcPYLs* ranged from 546 to 669 bp (Table 2). On the other hand, WolfsPort’s predicted subcellular localization of *EcPYL*s genes revealed that the majority of the putative *PYL*s genes of finger millet were located in the cytoplasm and chloroplast (Table 3). Further, all the putative *PYLs* of finger millet were hydrophilic in nature as they possess negative value of Grand Average of Hydropathicity.

**TABLE 2:** Gene position, ORF length, domain position and chromosome location of putative *PYLs* genes of finger millet.

NCBI Acc. no.	Transcript name	Gene name	Gene start (bp)	Gene end (bp)	Chromosome name	ORF length (bp)	PFAM ID	PFAM description	Domain position HMM SCAN
GJM92688.1	ELECO.r07.1AG0038470.1	*EcPYL1-1A*	51,299,793	51,300,389	1A	597	PF10604	Polyketide_cyc2	47–194
GJN17299.1	ELECO.r07.1BG0088430.1	*EcPYL2-1B*	66,773,446	66,774,072	1B	627	PF10604	Polyketide_cyc2	56–204
GJN19381.1	ELECO.r07.2AG0106820.1	*EcPYL3-2A*	5,737,836	5,738,444	2A	609	PF10604	Polyketide_cyc2	38–187
GJN19492.1	ELECO.r07.2AG0108140.1	*EcPYL4-2A*	6,745,596	6,748,419	2A	636	PF10604	Polyketide_cyc2	53–198
GJM91546.1	ELECO.r07.2AG0117770.1	*EcPYL5-2A*	16,588,979	16,589,524	2A	546	PF10604	Polyketide_cyc2	15–165
GJM90649.1	ELECO.r07.2BG0160340.1	*EcPYL6-2B*	5,566,370	5,566,972	2B	603	PF10604	Polyketide_cyc2	38–185
GJN19492.1	ELECO.r07.2BG0161780.1	*EcPYL7-2B*	6,714,154	6,716,983	2B	639	PF10604	Polyketide_cyc2	54–199
GJN20187.1	ELECO.r07.2BG0171370.1	*EcPYL8-2B*	14,790,778	14,791,404	2B	627	PF10604	Polyketide_cyc2	41–192
GJN08144.1	ELECO.r07.3AG0244730.1	*EcPYL9-3A*	45,026,141	45,026,809	3A	669	PF10604	Polyketide_cyc2	70–216
GJN31193.1	ELECO.r07.3BG0283350.1	*EcPYL10-3B*	54,043,193	54,043,861	3B	669	PF10604	Polyketide_cyc2	70–216
GJM88866.1	ELECO.r07.5AG0369710.1	*EcPYL11-5A*	6,215,159	6,215,770	5A	612	PF10604	Polyketide_cyc2	51–196
GJM94889.1	ELECO.r07.5AG0401550.1	*EcPYL12-5A*	59,625,111	59,626,595	5A	627	PF10604	Polyketide_cyc2	50–196
GJN14692.1	ELECO.r07.5BG0417310.1	*EcPYL13-5B*	5,193,046	5,193,648	5B	603	PF10604	Polyketide_cyc2	48–193
GJN35776.1	ELECO.r07.5BG0450090.1	*EcPYL14-5B*	74,809,940	74,811,451	5B	597	PF10604	Polyketide_cyc2	50–196

**TABLE 3:** Physicochemical attributes of putative PYL receptor proteins of finger millet.

EcPYL receptors	NCBI accession no.	No. of amino acids	Molecular weight (dalton)	PI value	Richness in amino acid	Total number of negatively charged residues (Asp + Glu)	Total number of positively charged residues (Arg + Lys)	Instability index	Aliphatic index	GRAVY	Subcellular localization
EcPYL1-1A	GJM92688.1	198	21150.91	8.65	Val (V)	20	23	55.52	85.51	−0.078	Cytoplasmic
EcPYL2-1B	GJN17299.1	208	22221.21	9.03	Val (V)	20	25	51.94	83.32	−0.079	Chloroplast
EcPYL3-2A	GJN19381.1	202	21950.57	6.55	Val (V)	24	22	41.5	76.63	−0.361	Cytoplasmic
EcPYL4-2A	GJN19492.1	211	23667.91	6.25	Val (V)	33	30	49.22	87.68	−0.394	Cytoplasmic
EcPYL5-2A	GJM91546.1	181	19497.12	6.2	Ala (A)	21	19	43	92.15	−0.117	Chloroplast
EcPYL6-2B	GJM90649.1	200	21688.29	6.38	Val (V)	24	22	45.97	76.9	−0.335	Cytoplasmic
EcPYL7-2B	GJN19492.1	212	23696.91	6.08	Val (V)	33	29	49.62	87.26	−0.39	Cytoplasmic
EcPYL8-2B	GJN20187.1	208	22277.03	5.5	Ala (A)	26	20	41.24	87.26	−0.218	Cytoplasmic
EcPYL9-3A	GJN08144.1	222	23204.15	6.58	Ala (A)	21	19	45.74	79.95	−0.05	Chloroplast
EcPYL10-3B	GJN31193.1	222	23181.11	6.54	Ala (A)	21	19	47.14	79.95	−0.052	Chloroplast
EcPYL11-5A	GJM88866.1	203	22052.87	8.72	Val (V)	21	24	44.1	84.98	−0.184	Chloroplast
EcPYL12-5A	GJM94889.1	208	22416.44	6.13	Val (V)	26	22	41.24	94.52	−0.128	Cytoplasmic
EcPYL13-5B	GJN14692.1	200	21586.24	8.36	Val (V)	21	23	47.94	83.3	−0.2	Chloroplast
EcPYL14-5B	GJN35776.1	198	21414.34	6.3	Val (V)	25	22	38.38	90.96	−0.152	Cytoplasmic

### Gene structure and conserved motifs analysis

**FIGURE 2:**
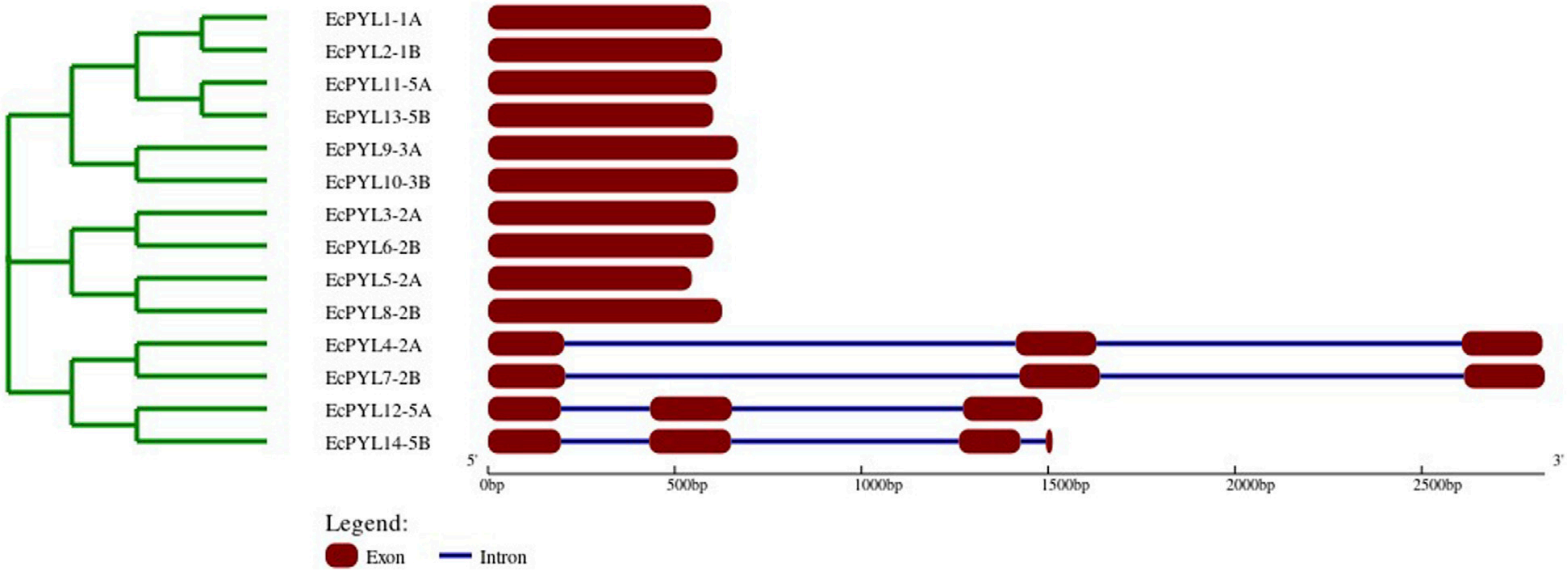
Evolutionary analysis and gene structure organizations of *EcPYL* genes, Exons and introns are represented by maroon boxes and blue lines, respectively. Scale represents the sizes of exons and introns.

**FIGURE 3:**
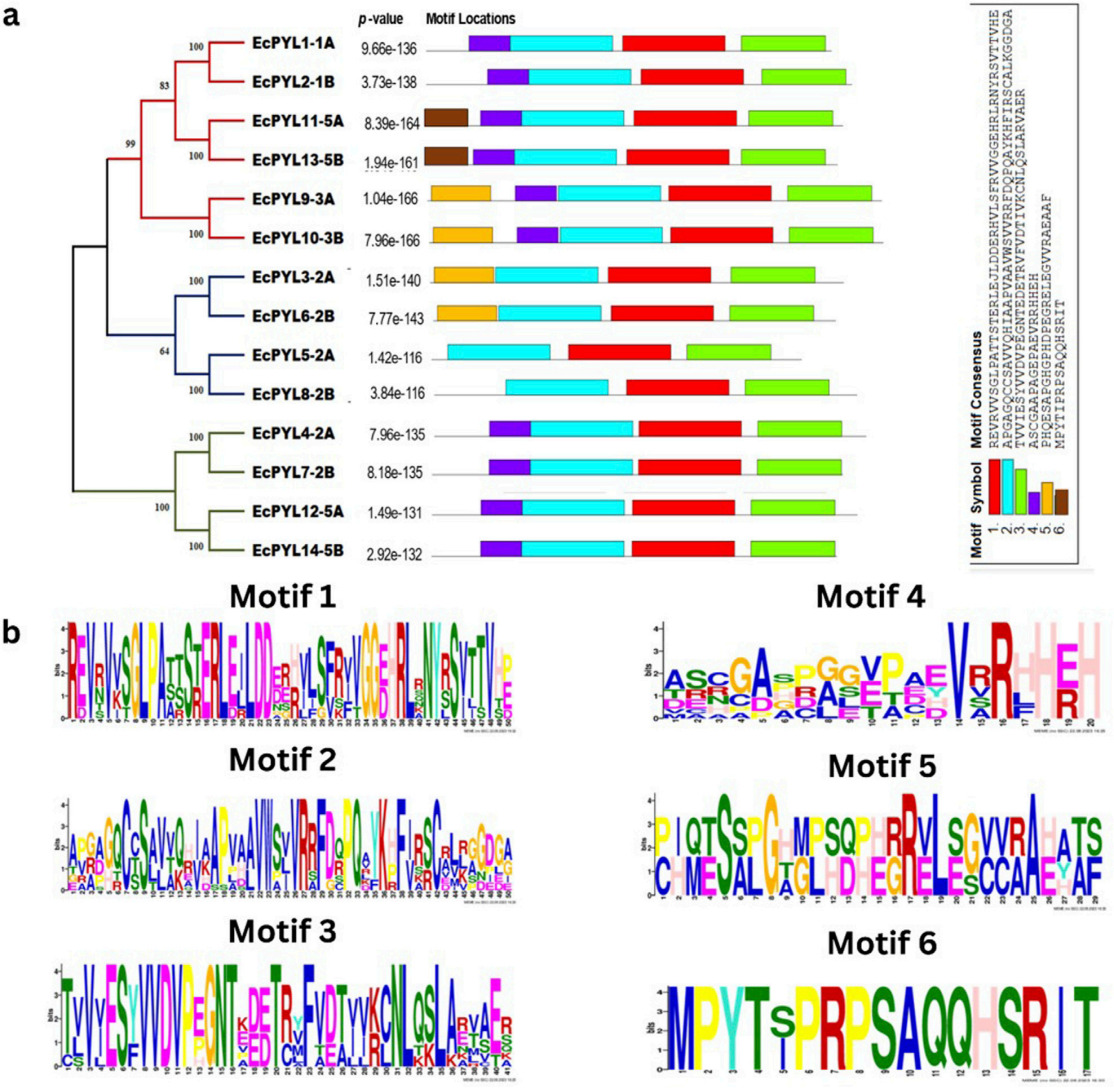
Evolutionary relation with reference to conserved motifs of *PYL* genes in finger millet. **(a)** evolutionary analysis classified *EcPYL* genes into three subfamilies, Subfamily-I (Red), subfamily-II (Blue) and Subfamily-III (Green), with their conserved motif. The different colours represent the 6 identified motifs. Motif 1-3 (red, cyan and green) are conserved across all 14EcPYLs, whereas motif 4 (blue) is conserved among subfamily I and III. Motif 5 (orange) is conserved for EcPYL-9-3A/10-3B/3-2A/6-2B of subfamily I and II), and Motif 6 (brown) is only present in EcPYL11-5A and EcPYL13-5B. **(b)** The six highly conserved sequence logos in *EcPYLs*. The sequence logos are the motifs which found to be conserved in 14 *EcPYL* proteins based on full-length alignments. The height of each letter in logo represents frequency of its occurrence at that site.

**FIGURE 4:**
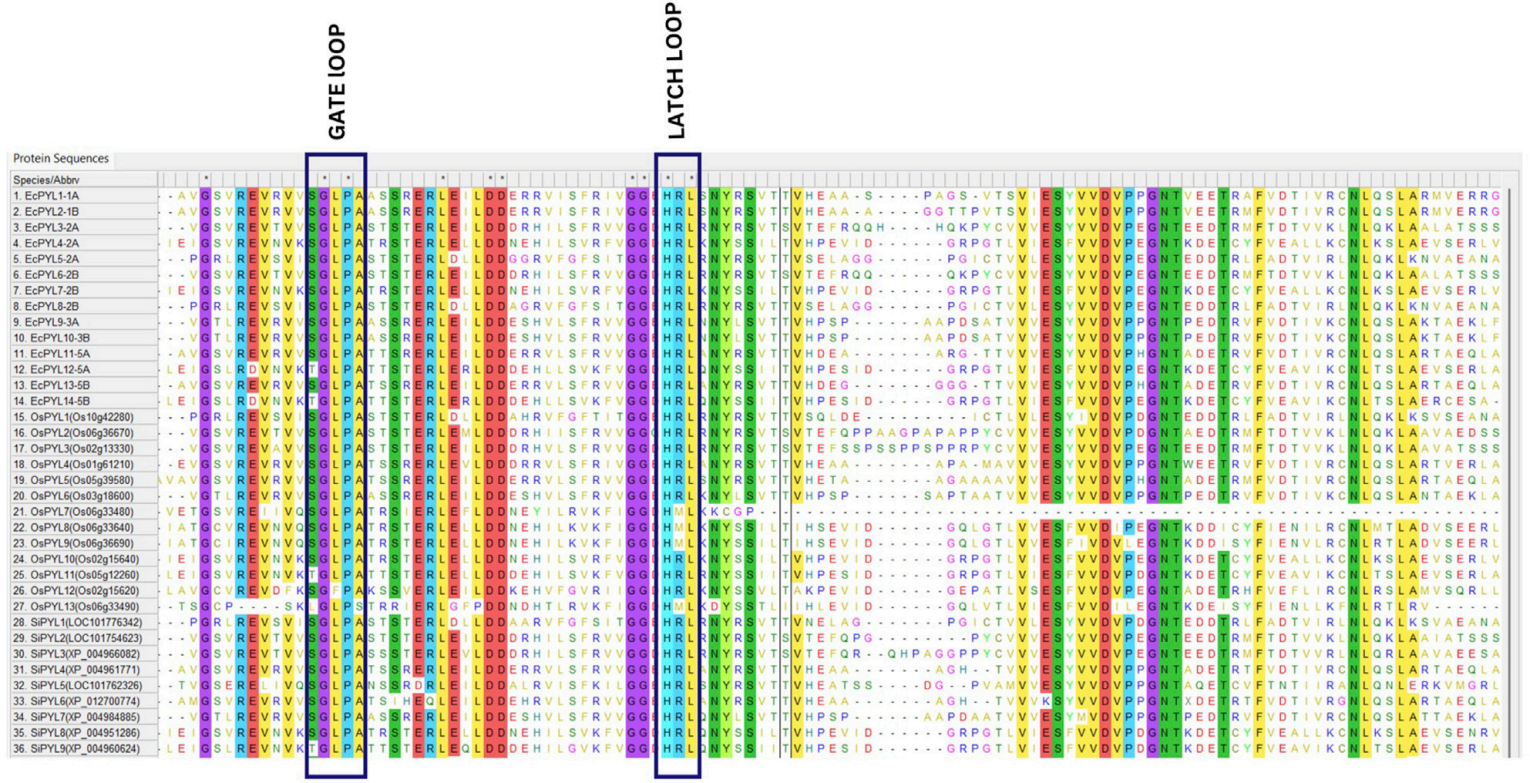
Multiple sequence alignment of *PYL* genes of finger millet, rice, and foxtail millet. MSA of putative *PYLs* genes of finger millet, rice and foxtail millets illustrate conserved GATE (CL2 loop) and LATCH (CL3 loop), essential for abscisic acid signaling.

It was demonstrated that *EcPYL* can be classified into two distinct clades based on the intron/exon structure analyzed using TB tool’s (Figure 2). In subfamilies I and II, no introns were detected, whereas all members of subfamily III except *EcPYL14-5B* contain two introns (Figure 2). As shown in Figure 3a, motifs 1, 2 and 3 were conserved across all *EcPYL* genes. Among the 14 *EcPYLs* studied, motif 1 contains the conserved characteristic Gate–Latch domain (Figure 4). However, motif 4 was found to be conserved across all receptors except *EcPYL3/5/8*. Motif 5 was found to be present only in the *EcPYL3/6/9/10* genes, whereas *EcPYL11* and *EcPYL13* of the 5A and 5B chromosomes contained Motif 6. The members of the subfamily share conserved motifs and possess unique motifs (Figure 3a). The unique motifs could indicate that each member performs a unique or specialized biological function, while similar motifs reveal functional similarities. Figure 3b displays the logos of these identified motif groups, indicating the frequency of amino acid occurrence at that site.

### Evolutionary analysis

**FIGURE 5:**
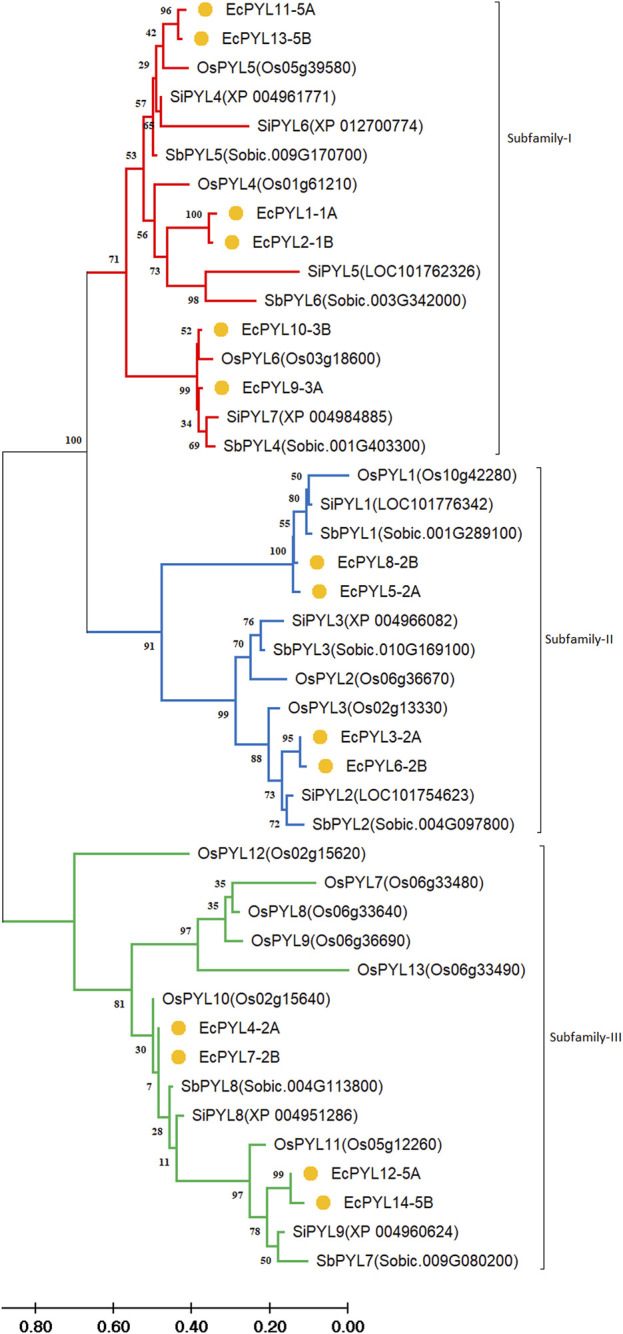
Comparative Phylogenetic analysis between finger millet, rice, foxtail millet and Sorghum. Tree was constructed by MEGA11 using Maximum Likelihood method with 1000 bootstraps Three subfamilies are represented in different colours: Subfamily‐I (Red), subfamily‐II (blue), and subfamily-III (Green). At the same time, the genes of finger millet throughout the phylogenetic are marked by orange nodes. The genes appear in the same clade of one subfamily having higher similarity, whereas all are evolutionarily connected. The branch length represents the magnitude of genetic change.

In terms of sequence similarity, *EcPYL* gene can be grouped in three major subfamilies, namely, subfamily-I, subfamily-II, and subfamily-III (Figure 5). Among the 14 *EcPYL* genes, *EcPYL1/2/11/13/9/10* (six *PYLs* distributed among chromosomes 1A,1B, 5A, 5B and 3A, 3B) belong to subfamily I; *EcPYL3/6/5/8* belong to subfamily II (four *PYLs* distributed among chromosomes 2A and 2B), while *EcPYL4/7/12/14* belong to subfamily III (four *PYLs* distributed among chromosomes 2A, 2B and 5A,5B). A comparative evolutionary tree (Figure 5) shows that the *PYL* genes of rice (13 *OsPYL*), sorghum (8 *SbPYL*), finger millet (9 *SiPYL*) and foxtail millet (14 *EcPYL*) are grouped together into subfamilies I, II, and III. The genes *EcPYL11/13* in subfamily I are closely related to one another and distantly related to *OsPYL5*, suggesting that these genes might be involved in signaling pathways that help plants resist the rice blast fungus (Yadav et al., 2020)*.* Along with this, *EcPYL10* may also play a similar role because it is closely related to *OsPYL6* and has the same inhibitory phosphatase activity (Kim et al., 2014). *EcPYL9* is closely related to *SiPYL7/4* and also has some evolutionary similarities with *EcPYL10-B* from finger millet and *OsPYL6* from rice. These evolutionary similarities are made up of conserved amino acid residues. *EcPYL3/6* belong to subfamily II of the evolutionary tree and are closely related to foxtail millet *SiPYL2* as well as to sorghum *SbPYL2. EcPYL4/7* are distantly related to rice *OsPYL10*, suggesting that *EcPYL4/7* could inhibit phosphatase activity without ABA (He et al., 2014). In addition, the *OsPYL7/8/9/13* appear in different subclades (Figure 5) as a result of mutations in conserved amino acid residues of the latch loop (HRL), which are replaced by HML amino acid residues (Figure 4) in the same evolutionary tree.

### Non-synonymous (Ka)/synonymous (Ks) analysis

**FIGURE 6:**
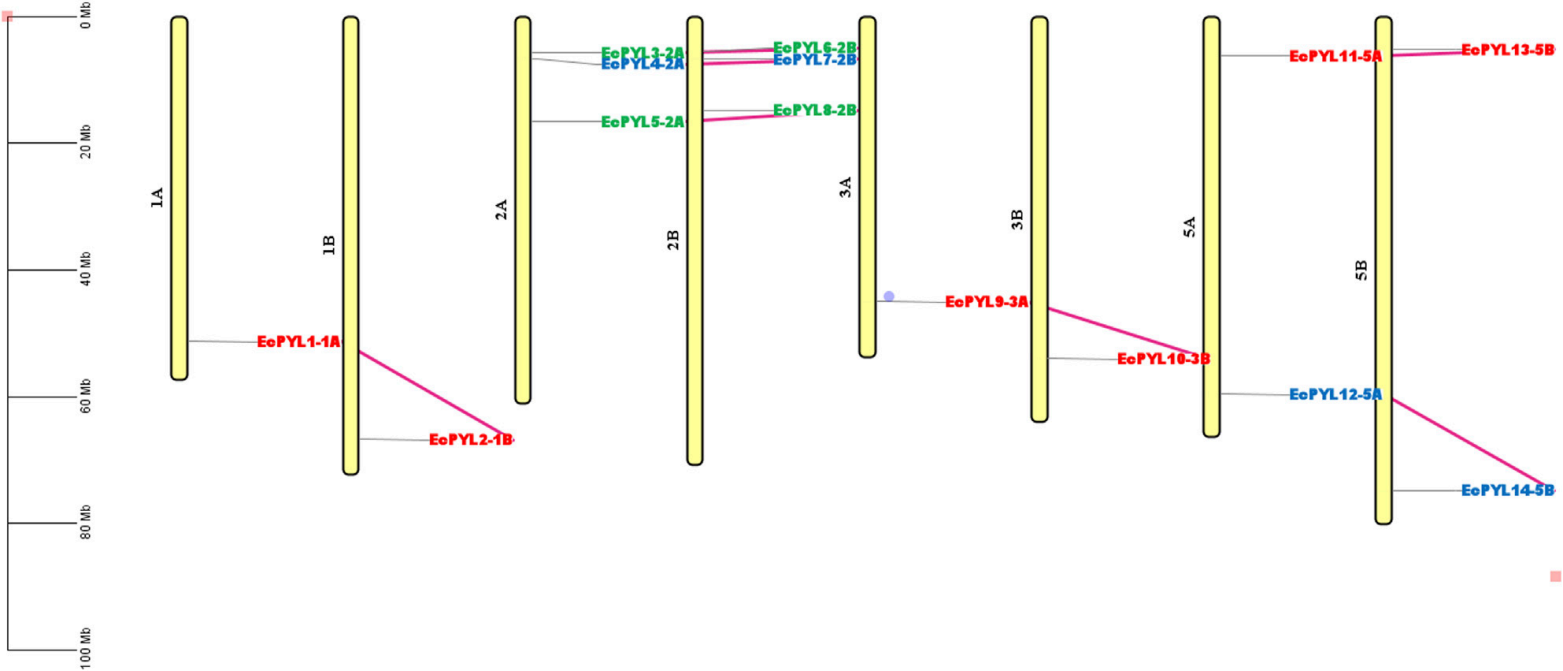
Physical mapping of homologous genes in finger millet genome. The labelled *EcPYL* genes in different colours represent different subfamilies; genes labeled with red belong to Subfamily‐I, blue belong to subfamily‐II, and green belong to subfamily‐III. The violet connecting line represents a homologous pair. At the same time, the scale represents the actual length of the chromosome.

To determine the degree of selection pressure during the divergence of *PYL* genes, we evaluated the (Ka) versus (Ks) analysis for the paralogous gene pairs of finger miller PYL receptor protein. Based on the Ka versus Ks calculation tool, seven pairs of paralogous genes with their position on the chromosome were identified for finger millet PYLs proteins, as shown in Table 4 and Figure 6. The selective pressure on genes due to which encoded protein shows functional alteration can be predicted by nucleotide substitution rate, i.e., the ratio of Ka (non-synonymous) versus Ks (synonymous) (Table 4). All gene pairs with Ka/Ks < 1 indicate the possibility of synonymous substitution and stabilizing selection during evolution. Moreover, the evolutionary time study of these paralogous genes revealed that they evolved approximately 3.1 million years ago.

**TABLE 4:** Ka vs. Ks estimation of putative *PYLs* gene of finger millet.

Gene pair	Ka	Ks	Ka/Ks ratio	Time (MYA)	Type of selection
*EcPYL13-5B, EcPYL11-5A*	0.009790675	0.04479112	0.2185852	3.44	Stabilizing selection
*EcPYL2-1B, EcPYL1-1A*	0.016148925	0.03464561	0.46611756	2.66	Stabilizing selection
*EcPYL10-3B, EcPYL9-3A*	0.013856485	0.05121111	0.27057576	3.93	Stabilizing selection
*EcPYL6-2B, EcPYL3-2A*	0.00657959	0.0474591	0.13863707	3.65	Stabilizing selection
*EcPYL8-2B, EcPYL5-2A*	0.01085052	0.03981233	0.2725417	3.06	Stabilizing selection
*EcPYL7-2B,EcPYL4-2A*	0.001426055	0.04195365	0.0339912	3.22	Stabilizing selection
*EcPYL12-5A,EcPYL14-5B*	0.007414455	0.02086289	0.35538964	1.60	Stabilizing selection

### Cis-regulatory elements (CRE) analysis

**FIGURE 7:**
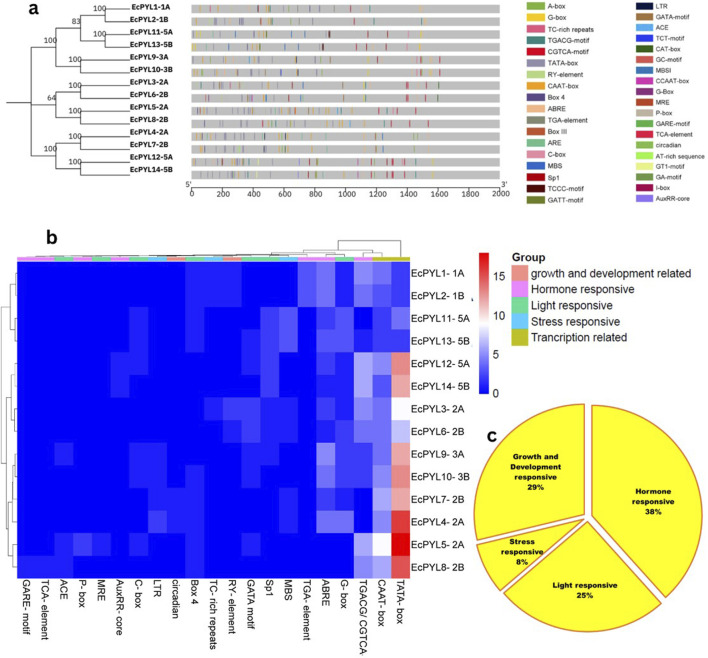
The *Cis*-regulatory element (CRE) analysis of *EcPYL* gene promoters. **(a)** The locations of CREs in the 14 *EcPYL* gene promoters. The grey rectangular horizontal bar represents the 2 kb promoter regions, and the different color boxes correspond with the different kinds of CREs. The horizontal values represent the position of CRE in 2KB promoter length. **(b)** A heatmap of CREs in the 14*EcPYL* gene promoters. The different colour boxes indicate the number of CREs in different *EcPYL* gene promoters. The dark blue colour boxes represented no corresponding CRE, and the red boxes represent eighteen corresponding CREs (highest number). The phylogenetic tree present at upper side of heat map showing 5 colour box represent different groups of the CRE responsible for specific functions. Whereas left side of tree represent subfamily of *EcPYL* genes. **(c)** Pie chart represent abundance of CRE in promoter of *PYL* genes of finger millet playing role in hormone response, stress response, light response and plant growth and development in *EcPYL* genes

CREs in the promoter region are known to influence transcriptional regulation greatly. Across fourteen *EcPYL* genes, 37 putative CREs were identified and mapped on the promoter region of *EcPYL* genes (Figure 7a; Supplementary Table S2). Several cis-elements were found to have different functions in finger millet. These included role in hormonal signaling (ABRE, TGACG/CGTCA-motif, P-box, GARE-motif, AuxRR-core TGA-element, and TCA-element), developmental processes (RY-element and circadian), light-responsiveness (G-box, C-box, Box 4, ACE, GATA motif MRE and Sp1) and various stresses (TC-rich repeats, LTR, MBS) in finger millet (Figure 7b). In addition to transcription-related CRE, a lot of the ABREs (abscisic acid-responsive element) and MeJA (methyl jasmonate/TGACG-motif/CGTCA-motif), which are linked to plant defense systems, were found. Furthermore, the fact that several *EcPYL* genes have GARE (gibberellic acid-responsive elements) and ARE (auxin-responsive elements) in their promoter region suggests that hormones may interact at the level of ABA receptor expression. Among stress-responsive factors, MBS (MYB binding site involved in drought-inducibility) and LTR (low-temperature responsive elements) are commonly observed in finger millet. Further, one additional CRE has been observed to be ubiquitously present in all the *PYL* genes of finger millet, called CCAAT. This five nucleotide base motif is associated with controlling flowering in plants (Wenkel et al., 2006) and contains a binding region for Nuclear Factor-Y transcription factor (NF-Y), which has been reported to regulate several biotic and abiotic stress factors (Rani et al., 2023; Rani et al., 2024a; Rani et al., 2024c). Hence, the presence of different CREs (8% stress responsive, 25% light responsive, 29% growth and development related and 38% hormone responsive) in the promoter region of finger millet provides an insight into its putative role in plant development and stress tolerance (Figure 7c).

### Protein-protein interaction and *EcPYL-*miRNA interaction analysis

**FIGURE 8:**
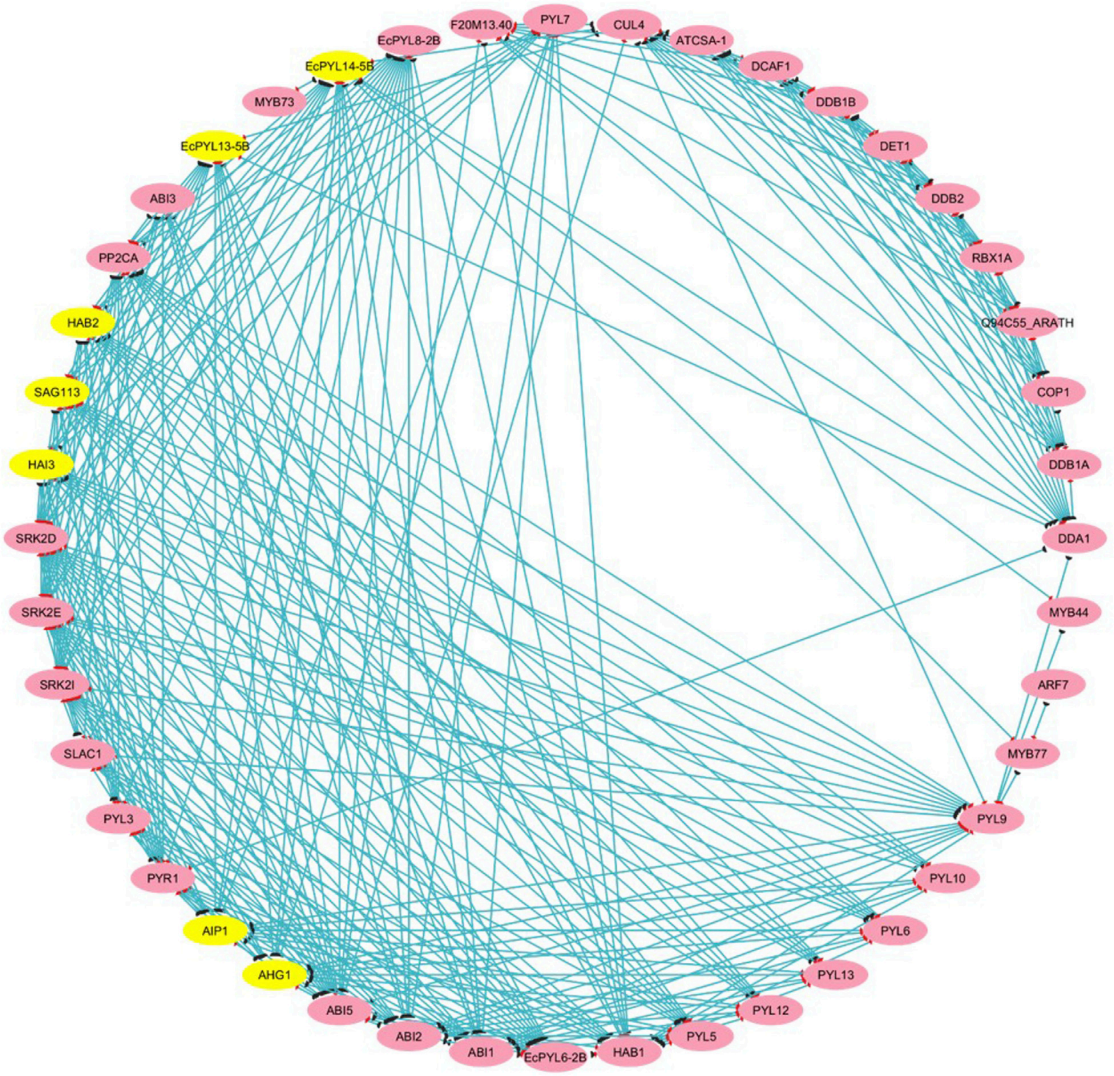
Protein-Protein (P-P) interaction analysis between putative PYLs of finger millet. The P-P interaction network is drawn using STRING tool and visualized using Cytoscape v. 3.10.3. The yellow highlights represent major genes that participated in interaction with other proteins or transcription factors that appeared in clusters analyzed through MCODE. At the same time, pink highlights represent (targets). The blue line represents the interacting string.

**FIGURE 9:**
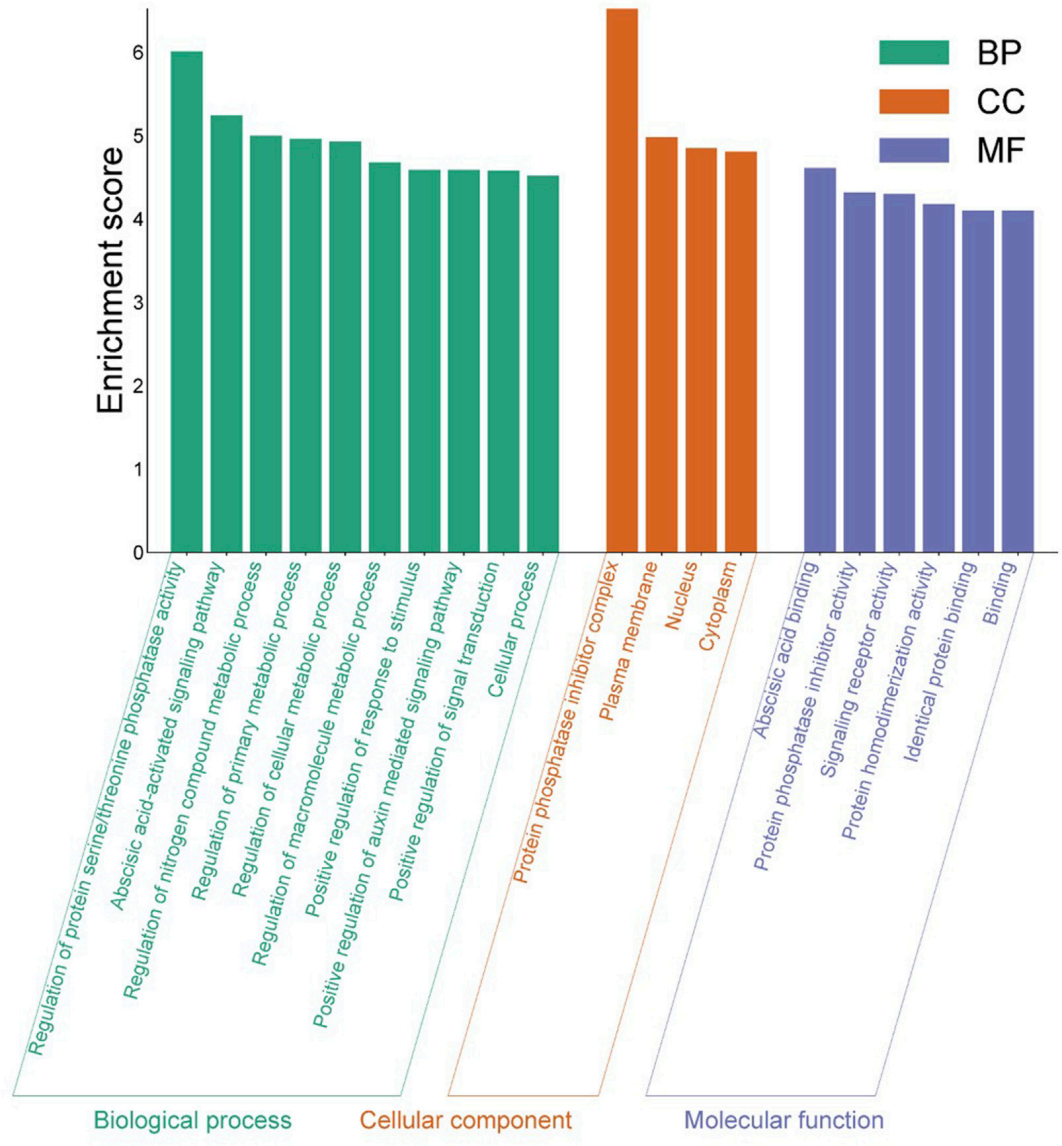
Gene Ontology analysis of identified *EcPYL* gene. The enrichment analysis shows the involvement of *EcPYL* in biological processes, molecular functions, and cellular components. In all, 20 GO terms were over-represented by > 4-fold enrichment value, with *p*-values <0.05.

The PPI results reveal a total of 44 nodes and 289 edges, and the MCODE of Cytoscope classifies them into three clusters. There are four *EcPYL* genes in cluster 3, and they interact with nine *Arabidopsis PYL* genes and 35 other genes performing the similar biological functions (Figure 8). In most cases, *EcPYLs* interact with PP2C, SnRK2 (the core ABA signaling complex), and the MYB transcription factor, which are vital components of the ABA-auxin signaling complex (Figure 8). Based on functional annotation, a group of EcPYLs proteins was discovered to play a role in different biological functions (BF), molecular functions (MF), cellular component (CC) (Figure 9).

Gene ontology and enrichment analysis reveals that *EcPYL* genes are enriched in plasma membrane (GO: 0005886), nucleus (GO: 0005634), protein phosphatase inhibitor complex (GO: 0062049) in terms of cellular components. Molecular function is enriched in abscisic acid binding (GO: 0010427), protein phosphatase inhibitor activity (GO: 0004864) and signaling receptor activity (GO: 0004864). Biological processes revealed that *EcPYL* genes are involved in regulating protein serine/threonine phosphatase activity (GO: 0080163) and the abscisic acid-activated signaling pathway (GO: 0009738). Besides these functions *EcPYL* genes also involved in two KEEG pathways *viz.* mitogen-activated protein kinase (MAPK) signaling pathway (ath04016) and plant hormone signal transduction pathway (ath04075).

**FIGURE 10:**
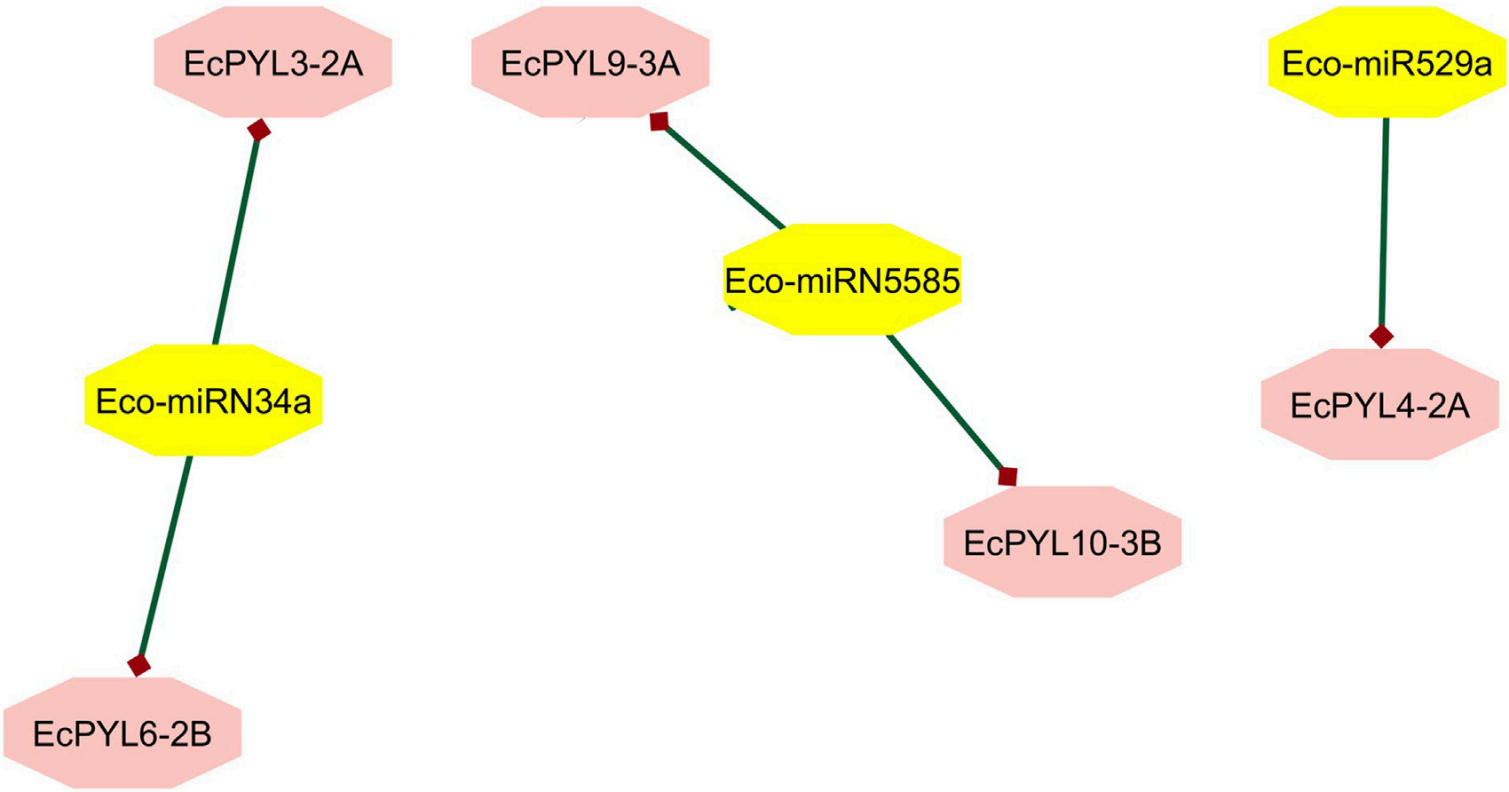
Finger millet, *EcPYL* genes, and miRNA interaction. *EcPYL-*miRNA interaction analysis carried out by using psRNA target and interacting network were visualized in cystoscape v. 10.3, reveals Eco-miRN5585 is targeting *EcPYL9-3A* and *EcPYL10-3B* through cleavage activity. Whereas, Eco-miRN34a is targeting two of EcPYL receptors protein EcPYL3-2A and EcPYL6-2B. Eco-miR529a is targeting *EcPYL4-2A* gene of finger millet.

In order to determine the miRNA targets and their regulation post-transcriptionally, EcPYL-miRNA interaction analysis was conducted, which revealed only three unique miRNAs targeting specific members of a particular subfamily of *PYL* genes in finger millet (Figure 10). Eco-miRN5585 targets *EcPYL9-3A* and *EcPYL10-3B* genes of subfamily I, while *Eco-miRN34a* targets *EcPYL3-2A* and *EcPYL6-2B* of subfamily-II and *Eco-miRN529a* targets only one gene *EcPYL4-2A* of subfamily III (Supplementary Table S3) through cleavage activity. Inhibition through cleavage activity appears to be the predominant mechanism by which most of the miRNAs predicted to target *EcPYL* members and regulating their expression post-transcriptionally either by positive or negative mechanism. However, miR529a were found to be involved in modulating panicle architecture in plants; however, miR5585 and miR34a were reported to play a crucial role as tumour suppressor cells (Farooqi et al., 2017). Thus, controlling *EcPYL* genes through sequence-specific interaction mediated by miRNAs could be very important for plants’ ability to respond to growth and pathogen infection.

### Pre-selection of candidate genes by ML algorithm

**FIGURE 11:**
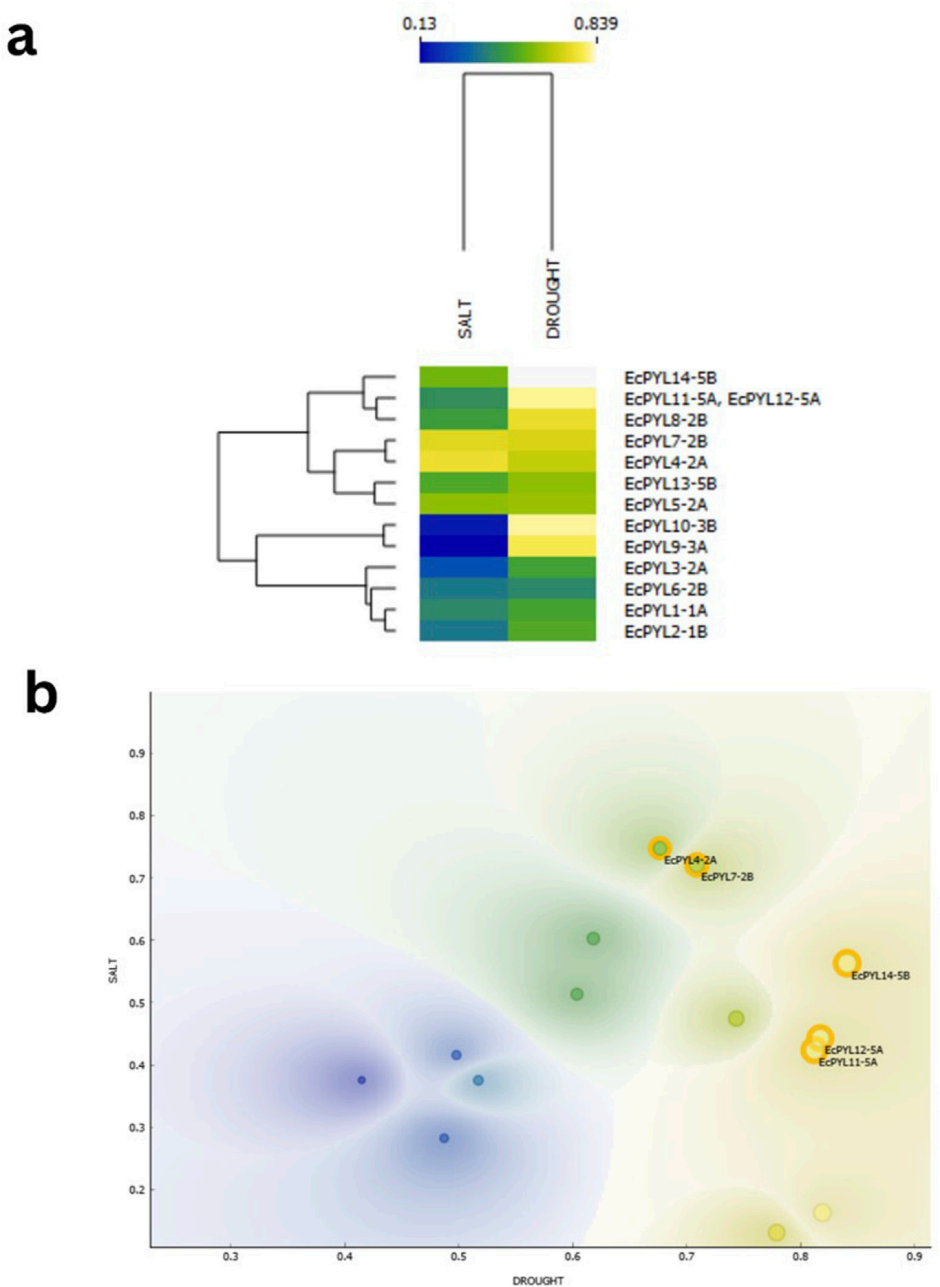
ML based prediction for expression of candidate genes under drought and salt stress represented by Heatmap and scatter plot. **(a)** Heatmap with hierarchical clustering illustrating the probability-based expression patterns of various genes under salt and drought stress conditions. The color scale represents expression probability values, ranging from 0.13 (blue) to 0.839 (yellow). Genes are clustered based on similarity in expression profiles, and their respective stress conditions (salt and drought) are indicated at the top of the dendrogram. **(b)** Scatter plot generated using Orange software, displaying the distribution of genes based on their expression probabilities under salt and drought conditions. Contour density shading represents regions of varying gene expression probability distributions. The best candidate genes, identified based on their significant expression in both conditions, are highlighted with orange circles. The x-axis represents drought stress probability, while the y-axis represents salt stress probability.

By combining scatter plot visualization (Figure 11b), hierarchical clustering (Figure 11a), and numerical probability assessments (Table 5), the expression probabilities of these genes were analyzed under both the stress conditions. Scatter plot analysis (Figure 11b) identified distinct clusters of *EcPYL* genes under salt and drought conditions, each exhibiting varying degrees of expression. *EcPYL14-5B*, *EcPYL12-5A*, *EcPYL11-5A* and *EcPYL7-2B* were most likely to be expressed during drought stress, which suggests they may be involved in mechanisms that help plants deals with drought. Conversely, *EcPYL4-2A* and *EcPYL7-2B* displayed the highest expression probabilities under salt stress (≥0.72), indicating their potential role in salinity adaptation. The hierarchical clustering heatmap (Figure 11b), which grouped genes with similar expression patterns, further supported these observations. Drought stress strongly linked the groups of *EcPYL14-5B*, *EcPYL12-5A*, and *EcPYL11-5A*. On the other hand, *EcPYL4-2A* and *EcPYL7-2B* grouped separately, indicating their role in salinity stress. Further, *EcPYL9-3A* and *EcPYL10-3B* showed a strong preference for drought stress, while *EcPYL6-2B* exhibited a lower expression probability under both conditions. Table 5 shows that these trends were supported by the probability analysis. *EcPYL14-5B* had the highest expression probability (0.839) during drought stress, followed by *EcPYL12-5A* (0.818) and *EcPYL11-5A* (0.811). The *EcPYL4-2A* (0.749) and *EcPYL7-2B* (0.721) were also among the most highly expressed genes under salt stress. Although some genes displayed moderate expression probabilities under both stress conditions, they did not meet the threshold for high likelihood (≥0.70). For example, *EcPYL5-2A* showed a fairly balanced expression pattern (0.604 when exposed to salt and 0.618 when exposed to drought), which suggests it might play a small but important role in adapting to abiotic stress in general. However, genes such as *EcPYL9-3A* (0.13 under salt, 0.781 under drought) and *EcPYL10-3B* (0.164 under salt, 0.819 under drought) showed a distinct preference for drought-related expressions. Overall, our results show that *EcPYL14-5B*, *EcPYL12-5A*, *EcPYL11-5A* and *EcPYL7-2B* are important candidate genes and these genes are likely to be involved in regulating drought stress. While *EcPYL4-2A* and *EcPYL7-2B* are likely to regulate salinity stress.

**TABLE 5:** Probability of expression of *EcPYL* genes under salt and drought stress conditions.

Gene	Probability of expression under salt	Probability of expression under drought
*EcPYL1-1A*	0.415	0.497
*EcPYL2-1B*	0.374	0.516
*EcPYL3-2A*	0.282	0.486
*EcPYL4-2A*	0.749	0.676
*EcPYL5-2A*	0.604	0.618
*EcPYL6-2B*	0.377	0.414
*EcPYL7-2B*	0.721	0.808
*EcPYL8-2B*	0.477	0.743
*EcPYL9-3A*	0.13	0.781
*EcPYL10-3B*	0.164	0.819
*EcPYL11-5A*	0.422	0.811
*EcPYL12-5A*	0.444	0.818
*EcPYL13-5B*	0.512	0.603
*EcPYL14-5B*	0.564	0.839

The table presents the predicted probability scores for the expression of *EcPYL* genes in response to salt and drought stress, as determined by the ASRpro web server. Genes with a probability score of ≥0.70 were highly likely to be expressed under the respective stress conditions.

### Expression profile of *PYL* genes in finger millet under drought and salinity stress conditions

**FIGURE 12:**
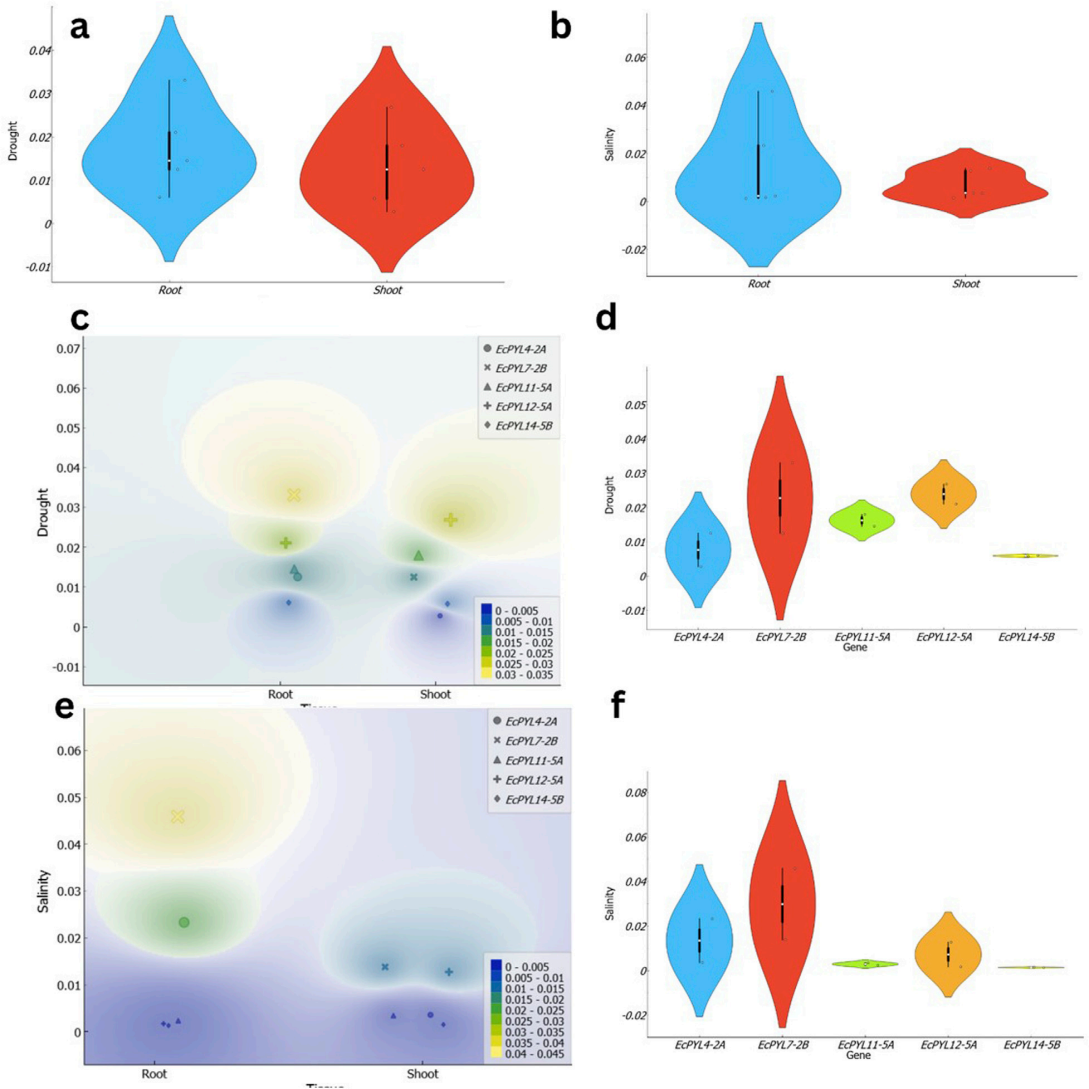
Illustration of the expression patterns of *EcPYL* genes in finger millet under drought and salinity stress across shoot and root tissues. **(a,b)** present violin plots depicting the distribution of *EcPYL* gene expression under drought and salinity stress, respectively. In **(a)**, the expression levels under drought conditions exhibit a broader distribution in root tissues (blue) compared to shoot tissues (red), suggesting higher variability in root responses. Similarly, in **(b)**, under salinity stress, root tissues display a wider expression range than shoot tissues, as indicated by the distribution density and black boxplots representing the median, interquartile range, and data spread. **(c,e)** depict density-based expression heatmaps of *EcPYL* genes under drought and salinity stress, respectively. In **(c)**, the density plot illustrates the spatial expression distribution of *EcPYL4-2A, EcPYL7-2B, EcPYL11-5A, EcPYL12-5A*, and *EcPYL14-5B* across root and shoot tissues under drought stress. The color gradient represents expression density, where yellow and green indicate high expression levels, while blue represents low expression levels. A similar pattern is observed in **(e)** under salinity stress, where root tissues exhibit a more pronounced variation in expression, while shoot tissues display a more localized expression response. **(d,f)** show violin plots representing the expression profiles of individual *EcPYL* genes under drought and salinity stress, respectively. Under drought stress **(d)**, *EcPYL7-2B* exhibits the highest variation, while *EcPYL14-5B* remains relatively stable across both tissues. Similarly, under salinity stress **(f)**, *EcPYL7-2B* again shows the highest expression variability, whereas *EcPYL11-5A* and *EcPYL14-5B* demonstrate limited fluctuations in expression levels.

Through ML based algorithm, a total of five *PYL* genes (*EcPYL4-2A, EcPYL7-2B, EcPYL11-5A, EcPYL12-5A*, and *EcPYL14-5B*) that respond to drought and salinity stresses were selected (Figures 11a,b), and their expression pattern was analyzed in shoot and root tissues of finger millet under drought and salinity stress (Figures 12a,b). Notably, all genes exhibited expression in both tissues under both the stress conditions (Figures 12a,b,d,f); however, their expression patterns varied between tissues and conditions (Figures 12c,e). For instance, compared to *EcPYL4-2A*, only *EcPYL12-5A* showed a 9.69-fold increase in shoot tissue under drought stress conditions (Figure 12c). When we compared the expression pattern of *EcPYL12-5A* in shoot tissue between salinity and drought, it was observed that drought stress increased its expression level up to 2.1-fold compared to salinity stress (Fig. d–f). The expression level of *EcPYL11-5A* was higher in the shoot after *EcPYL12-5A* under drought. Under drought stress, *EcPYL7-2B* increased more than 2-fold in shoot tissue compared to *EcPYL4-2A* and *EcPYL14-5B*. Three genes (*EcPYL4-2A*, *EcPYL11-5A*, and *EcPYL14-5B*) were comparatively less active in the shoot tissue of finger millet under salt stress (Figure 12e). However, of these, *EcPYL4-2A* increased its expression level by 0.76-fold higher in shoot tissue under salinity stress than drought (Figure 12f). Two genes (*EcPYL7-2B* and *EcPYL11-5A*) showed their expression level higher (>3.5-fold) in shoot tissues under salinity stress than the other three genes. In conclusion, three genes (*EcPYL7-2B*, *EcPYL11-5A*, and *EcPYL12-5A*) and two genes (*EcPYL7-2B* and *EcPYL12-5A*) were found to be highly expressed in shoot tissue of finger millet under drought and salinity stresses, respectively.

In the root, *EcPYL2-7B* exhibited highest expression among the five PYLs under both stresses. Notably, expression level of *EcPYL12-7B* increased by more than 15-fold compared to *EcPYL11-5A*, *EcPYL12-5A*, and *EcPYL14-5B* under salinity stress. Similarly, the same gene demonstrated 1.96-fold higher expression in the root under salinity stress compared to *EcPYL4-2A*. However, *EcPYL4-2A* expression was up to 9.3 times higher than *EcPYL11-5A*, 13.5 times higher than *EcPYL12-5A*, and 17.6 times higher than *EcPYL14-5B* in the root under salinity stress. Interestingly, the expression levels of the same three genes—*EcPYL11-5A*, *EcPYL12-5A*, and *EcPYL14-5B* was enhanced by 6.15, 12.26, and 4.6 times in root tissues under drought stress. Moreover, *EcPYL7-2B* alone had highest expression in the root under drought stress among the five genes. For instance, the expression level of this gene was almost 1.5 times higher than that of the other genes. Overall, *EcPYL4-2A* and *EcPYL7-2B* showed higher expression in the root tissue, under both the stress conditions suggesting that *EcPYL7-2B* is the most responsive gene under drought and salinity stress, particularly in root tissues, while *EcPYL12-5A* and *EcPYL11-5A* play significant roles in shoot tissues under drought stress. This differential expression highlights the potential role of these genes in stress adaptation and provides insights for future functional studies on drought and salinity tolerance mechanisms in finger millet.

### Homology modeling of finger millet *PYLs* genes

**FIGURE 13:**
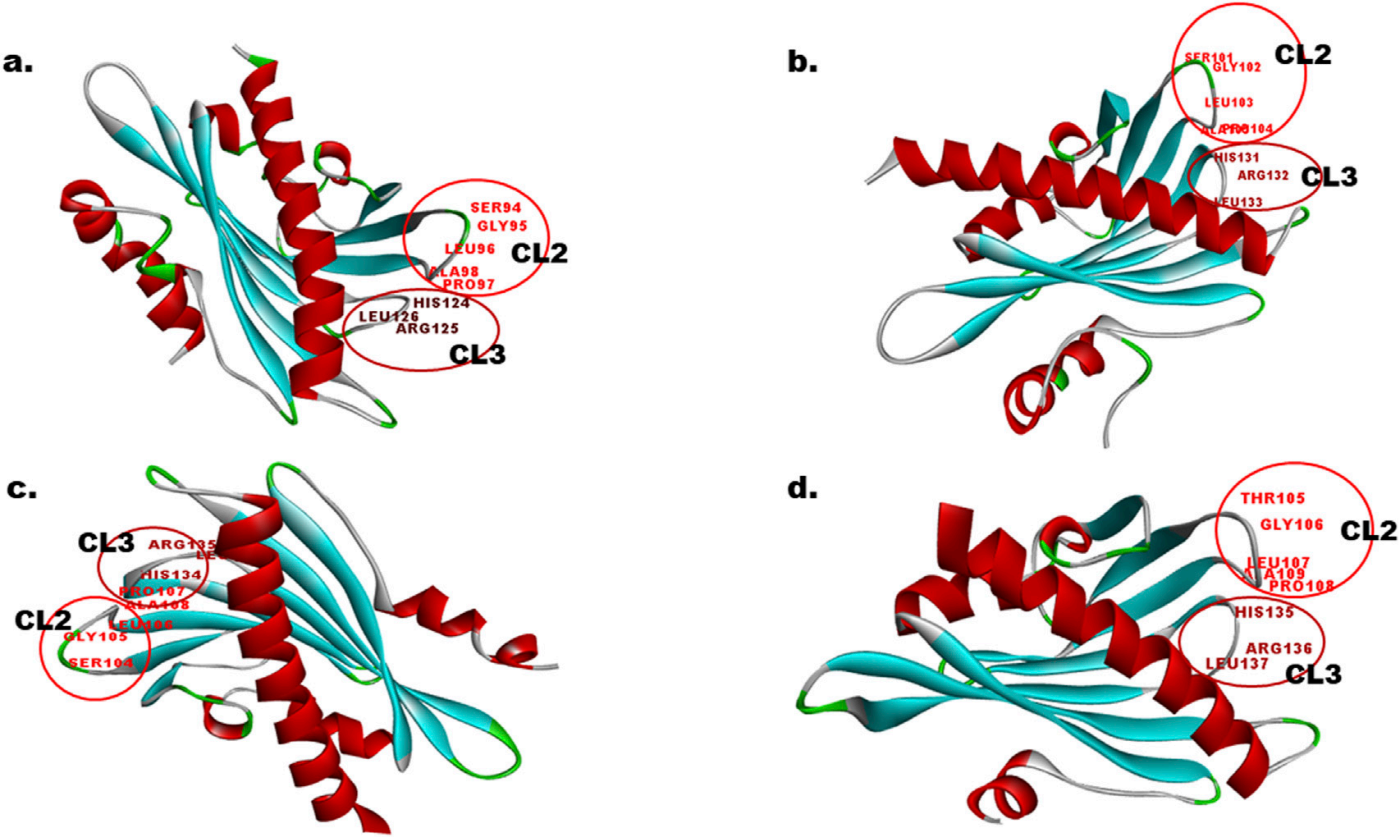
*In-silico* structural analysis of EcPYL proteins. The 3D structure modeling of EcPYL proteins was generated using the SWISS-MODEL and visualized by Biovia Discovery Studio. The alpha helix is represented by red colour, beta-sheets by cyan colour, coil by green and loops by gray colour. The conserved amino acids residue represented on loop (CL2 and CL3 loop) with their position, participating in ABA signaling **(a)** EcPYL6-2B, **(b)** EcPYL8-2B, **(c)** EcPYL13-5B, and **(d)** EcPYL14-5B.

Protein sequences, EcPYL13-5B, EcPYL6-2B, EcPYL8-2B, and EcPYL14-5B representing each clade of the phylogenetic tree, were used for homology modeling for structural prediction (Figure 5). The widely preferred method for modeling protein structures is to compare a sequence of our interest, i.e., the target sequence (unknown structure), with a template (known structure) and look for structural similarities. The SWISS-MODEL server (https://swissmodel.expasy.org/) was used in the present study for homology modeling of *EcPYL6-2B, EcPYL8-2B, EcPYL13-5B,* and *EcPYL14-5B* (Figure 13) against best-matched template 4oic, 5ujv and 5gwo, respectively, based on the high percent of identity, as shown in Table 5. The model generated by these templates was further validated by the SAVES server by analyzing the number of amino acid residues in allowed or disallowed areas through PROCHECK, accuracy of non-bonded contact by ERRAT (Table 6). All the models had ERRAT scores greater than the acceptable value of 80%. After the accuracy measurement, the modeled structure was subjected to the degree of similarity search with the crystal structure of (PDB ID 4oic, 5ujv and 5gwo) by using SuperPose V.1. The RMSD (Root Mean Square Deviation) and NRES (Number of Overlapped Residue) were analyzed for the superposed structure as shown in Table 4. By evaluating the result of the superposition, it can be concluded that the quality of the model is good, with the least deviation of residues from the template. Further, validation of the predicted model was performed with ResProx resolution criteria (Good: 0–1.5, Middle: 1.5–2.5, Bad: >2.5), indicating the models to be of good resolution. Table 6 summarizes the detailed analysis of homology modeling using SWISS-MODEL, structural assessment, SAVES server validation, and quality and resolution estimation using SuperPOSE v.1 and ResProx. The 2-D structural analysis performed by the PDBSum server revealed that finger millet *EcPYLs* genes (*EcPYL6-2B, EcPYL8-2B, EcPYL13-5B*, and *EcPYL14-5B*) contains same number of strand but having different number of helices as 5:4:5:4 respectively (Table 6). Figure 13 depicts each model’s conserved residues present in CL2 (GATE loop) and CL3 (LATCH loop), specific for ABA binding.

**TABLE 6:** Homology modelling, assessment, and validation of putative *EcPYLs* gene of finger millet, participated in Protein-Protein interaction.

Homology modeling				Structural assessment and validation				
Genes	Template PDB ID	Identity	Q MEAN D	Residues in most favoured regions	Residues in additional allowed regions	Residues in generously allowed regions	Residues in disallowed regions	ERRAT
*EcPYL6-2B*	4oic.1.A	77.72%	0.87 ± 0.06	92%	6.70%	1.20%	0.00%	81.6
*EcPYL8-2B*	5ujv.1.A	83.68%	0.87 ± 0.06	95%	4.90%	0	0	82.69
*EcPYL13-5B*	5ujv.1.A	55.56%	0.72 ± 0.07	95%	4.90%	0	0	84.67
*EcPYL14-5B*	5gwo.1.B	78.40%	0.86 ± 0.07	88.90%	11.10%	0	0	87.4

Quality and resolution estimation					2-Dimensional structural analysis						
Template resolution	Resolution of predicted model	RMSD	NRES	Helix		Strand	Beta turn	Beta hairpins	Helix-helix interacs	Gamma turn	Beta bulges
2.1	1.5	0.64	0.9	5		7	13	5	3	2	6
1.3	1.1	0.5	0.51	4		7	14	5	1	1	7
1.3	1.3	1.53	1.32	5		7	12	5	2	1	5
2	1.3	0.38	0.35	4		7	12	5	1	2	4

## Discussion

The *PYL* gene family is found in a variety of plant species, including *Arabidopsis* (Gonzalez-Guzman et al., 2012), rice (Kim et al., 2012; Yadav et al., 2020), sorghum (Dalal and Inupakutika, 2014), maize (Fan et al., 2018; Wang et al., 2018) and wheat (Lei et al., 2021). Rani et al. (2024c) reported three *EcPYL* genes (GJN19492.1, GJN35776.1, GJN19381.1) in finger millet based on rice homologs. However, the in-depth characterization and molecular validation of PYL receptors is yet to be explored in finger millet. A total of 14 *EcPYL* genes were identified in finger millet in the present study, which are distributed into three subfamilies designated as subfamily I, II and III respectively. Evolutionary analysis coupled with MEME search revealed a diversity of conserved motifs in finger millet and other cereals *PYL* genes. Whereas, the gene structure analysis showed presence of a common exon or intron structure in most of the genes within a subfamily. The members of *EcPYLs* in Subfamilies I and II are intronless and have conserved motifs 1, 2, and 3. However, only Subfamily III members contain introns and are conserved for motifs 1, 2, 3 and 4. Whereas motif 5 was observed among members of subfamily 2, and motif 6 was found only among two members of subfamily I. *EcPYL* genes in the same subfamily have gradually developed different protein motif structures due to adaptation to complex environmental conditions during the evolution of the finger millet genome. As compared to 14 *PYLs* in finger millet, there exist 13 *PYLs* in rice (Yadav et al., 2020), 8 *PYLs* in sorghum (Dalal and Inupakutika, 2014) and 9 *PYLs* in foxtail millet (Wu et al., 2023). The *EcPYLs* revealed close genetic proximity with rice and divergence with sorghum and foxtail millet *PYLs*. The variability could be attributed to gene duplication events resulting from polyploidization, which contribute to gene family expansion and genome evolution (Moore and Purugganan, 2005).

The chromosomal location of these *EcPYL* genes were determined and it was found that it exists as seven pairs of homologous genes present on homologous chromosomes 1, 2, 3, and 5. Most of these genes are located on chromosomes 2A and 2B. As a result of genome polyploidization, some homologous genes might have lost (Lynch and Force, 2000). Gene duplication plays an important role in expanding the *PYL* gene family with possibility for new biological functions (Conant and Wolfe, 2008). The analysis of cis-regulatory elements indicated that *EcPYLs* is associated with diverse roles such as hormone-responsive, light-responsive, stress-responsive, growth, development-responsive, and transcription-responsive genes. It has been reported that over-expression of *PYL* genes in *Arabidopsis* enhances the plant’s sensitivity to ABA during seed germination, growth, stomata regulation, and drought tolerance (Ma et al., 2009). Moreover, Rice ABA receptor OsPYL5 regulates ABA signaling, thereby improving drought and salinity tolerance and ABA sensitivity during germination and seedling development (Kim et al., 2012; Kim et al., 2014). Based on this analysis, it is evident that *PYL* genes may regulate finger millet growth and development and are actively involved in stress responses. Additionally, the presence of CCAAT cis-acting element in its promoter region indicates its involvement in regulation of flowering and providing binding site for nuclear factor-Y (NF-Y) transcription factor (Wenkel et al., 2006; Muthamilarasan et al., 2014; Rani et al., 2023). Further, this NF-Y-PYL/PYR module could regulate the expression of the ABA receptor and enhance ABA signal transduction via transcriptional regulation of PYR1 (Yu et al., 2021). This, in turn, promotes the expression of stress-responsive genes against drought and salt stress (Yu et al., 2021). Finger millet, extremely tolerant to drought and salt (Gupta et al., 2017; Antony Ceasar et al., 2018; Sood et al., 2019; Ceasar, 2022; Rani et al., 2023), possesses fourteen *PYLs* genes and shows an interaction network with several TFs (Figure 8). ABA has been found to play a crucial role in responding to abiotic stresses such as drought and salinity and ABA signaling is initiated by PYLs (Rani et al., 2024c). Many plants have been reported to have differential expression patterns of *PYL* genes within their tissues. Soybean seeds express many *PYL* genes at a relatively high level compared to other soybean tissues (Bai et al., 2013). The rubber tree, the transcript abundance of *PYL* genes is high in latex (Guo et al., 2017). Similarly, in rice *PYLs* genes are expressed at higher levels in root tissue under drought stress than in other tissues (Yadav et al., 2020). Under drought and salinity stress, finger millet exhibits a significant ABA response, but specific PYL responsible for this is still not deciphered. In the present study, *EcPYL7-2B* demonstrated their role in regulating both drought and salinity stress. Moreover, some of the genes like *EcPYL14-5B* of finger millet were found to be comparatively less expressed as compared to other genes under drought and salinity stress, which is in accordance with previous studies reported in sorghum, rice, grapes and tomato (Boneh et al., 2012; González-Guzmán et al., 2014; Yadav et al., 2020). This behavior may be explained by a negative feedback regulatory mechanism which indicates that when excessive amounts of ABA accumulate in leaves under stress, PYL expression may be inhibited (Merlot et al., 2001; Santiago et al., 2009) or the function of these genes may not be required for drought and salinity stress (Di et al., 2018). Based on the expression profiling results coupled with stress-responsive cis-elements in *EcPYL* promoters, it appears that some *EcPYLs* are responsive to drought and salinity. Hence, these PYLs may be potential candidate to enhance finger millet tolerance to abiotic stress.

An extensive network of interactions between PYL proteins of finger millet and *Arabidopsis* genes has been predicted. As a major component of the ABA signaling pathway, PYL interacts with SnRK and PP2C in finger millet (Figure 8). PP2CA inhibits ABA metabolism, essential to seed germination and cold tolerance (Romero et al., 2012). In guard cells that recognize ABA, ABI1 modulates both calcium channel permeability and actin reorganization within stomatal closure (Tähtiharju et al., 2002). It has been found that several finger millet *PYL* genes interact with PP2CA and ABI1, suggesting its possible role in seed germination and vernalization. In response to auxin stimulation, PYL8-MYB77 interaction enhances transcription activation of IAA19 in *Arabidopsis* (Shin et al., 2007). Similarly, it was observed that *EcPYL14-5B* interacts with MYB77 (Shin et al., 2007) and MYB44 (Qin et al., 2022), indicating that both proteins participate in ABA-auxin signaling and show multiple stress tolerance. Further, to decipher the relation between ABA signaling and miRNA regulation, a PYLs-miRNA interaction study was performed. The interaction between PYLs and miRNA reveals that *miRNA5585* targets *EcPYL10-3B* and *EcPYL9-3A*, while *miRNA34a* targets *EcPYL3-2A* and *EcPYL6-2B,* as shown in Supplementary Table S3. In contrast, *EcPYL4-2A* was targeted by *miRNA529a*, suggesting that *EcPYL4-2A* are involved in a complex regulatory network and might be associated with the panicle architecture (Yue et al., 2017). The protein interaction networks and miRNA-mediated pathways of *PYLs* genes elucidated in the present study might reveal a better understanding of the ABA signal transduction pathway in finger millet.

The structural studies attempted revealed that PYR/PYL belong to the Bet v superfamily, which contains the Bet v fold or START/polypeptide cyclase2 domain (Radauer et al., 2008). A beta-sheet with seven strands flanked by two alpha helices is referred to as a helix-grip fold. As an additional characteristic of this family of ABA receptors, the PYR/PYL family contains an alpha helical segment at their N-terminus, which is not found in the Bet v fold. Therefore, this segment can be considered as a typical characteristic of this family of ABA receptors (Santiago et al., 2012). Based on the crystallographic structure analysis of *Arabidopsis PYL* genes, it is evident that this gene contains a seven-stranded beta-sheet flanked by two alpha-helices (α1 and α2) and an additional alpha-helical segment (α_0_) at its N terminus. These alpha helices and beta sheets are connected with four conserved loops: CL1 connecting (α1 and β2), CL 2 connecting (β3 and β4), CL3 connecting (β5 and β6), and CL4 connecting (β7 and α2) (Yin et al., 2009). The *in silico* homology modeling attempted for the four PYLs of finger millet *EcPYL6-2B, EcPYL8-2B, EcPYL13-5B,* and *EcPYL14-5B* revealed seven strands flanking with two alpha helices with the presence of additional alpha helices at N-terminal. This indicates ABA receptor family having START domain. As shown in Figure 9, the helices and strands possess conserved CL1, CL2, CL3 and CL4. As reported for *Arabidopsis* crystal structure (PDB ID 4oic, 5ujv and 5gwo), the CL2 loop are conserved for Ser(S), Gly(G), Leu (L), Pro(P), Ala(A) amino acid residues whereas, CL3 loops are conserved for His (H), Arg (R) and Leu (L) amino acid residue (Yin et al., 2009). The constructed models of *EcPYL* showed conserved amino acids in the CL2 and CL3 loops (Figure 9), except *EcPYL14-5B*, where Ser (S) gets replaced by Thr (T) at the CL2 loop. Similar substitution has also been observed in maize *PYL* genes, which contain a conserved GATE loop or CL2 loop (SGLPA replaced with TGLPA amino acid residue) (Wang et al., 2018; Yadav et al., 2020). Hence, *PYL* genes in finger millet possess a conserved CL2 loop, which plays a vital role in ABA binding (Yin et al., 2009) and provides valuable information regarding their biological function. In order to achieve a high standard of quality, the model should contain over 90% of the residues in the most allowed regions (Laskowski et al., 2018). The model structure of this study indicates that 92%–95% of residues are mostly in allowed regions (Supplementary Figures S1–S4; Table 5), indicating that the predicted models can be accepted. The predicted structural insights provide an opportunity for deciphering the putative functions of EcPYL receptors in finger millets, which could be explored for crop improvement after validation through extensive wet-lab based experimentations.

## Conclusion

Finger millet genome mining for deciphering the putative *PYL* genes has been attempted in the present study. A total of 14 identified *EcPYL* genes have been extensively characterized *in silico* for multiple structural and functional properties. Chromosomal location, exon-intron structure, motif evaluation, and cis-regulatory element analysis and evolutionary analysis have been performed, revealing that the *EcPYLs* gene of finger millet is structurally and evolutionarily conserved among the three subfamilies. The presence of various cis-regulatory elements in the *EcPYLs* of finger millet may be involved in the enhancement of various stresses and developmental processes. Five genes (*EcPYL4-2A, EcPYL7-2B, EcPYL11-5A, EcPYL12-5A* and *EcPYL145B*) were found to be respond to drought and salinity stresses through machine learning approaches. Gene expression analysis revealed that under salinity and drought stress conditions, two key genes *EcPYL7-2B* and *EcPYL12-5A* were highly induced in shoot and root tissues. Further targeting of these two genes by genome editing approaches will help to identify the exact role of these genes in finger millet, which will aid in further breeding program. In addition, several CREs were found in this study from the promoter regions of *EcPYLs* of finger millet. Targeting drought and salinity stress responsive CREs using CRISPR activators has the potential to influence the expression of the downregulated *EcPYLs* genes. These significant findings could be applied for crop improvement programs by providing an insight into ABA signaling pathways associated with stress tolerance mechanisms in finger millet.

## Data Availability

The datasets presented in this study can be found in online repositories. The names of the repository/repositories and accession number(s) can be found in the article/Supplementary Material.

## Supplementary material

SUPPLEMENTARY FIGURE S1 PROCHECK analysis of EcPYL6-2B.

SUPPLEMENTARY FIGURE S2 PROCHECK analysis of EcPYL8-2B.

SUPPLEMENTARY FIGURE S3 PROCHECK analysis of EcPYL13-5B.

SUPPLEMENTARY FIGURE S4 PROCHECK analysis of EcPYL14-5B.

SUPPLEMENTARY Table S1 Retrieved PYL protein sequence (27) after BLASTP search against phytozome database in Finger millet genome.

SUPPLEMENTARY Table S2 Cis regulatory elements in promoter region of *EcPYLs* genes.

SUPPLEMENTARY Table S3 Putative miRNA and their targeted *PYLs* genes in finger millet.

## Abbreviations

ABA, Abscisic acid; PYR1, Pyrabactin Resistance 1; PYL, Pyrabactin Resistance 1-like; RCAR, Regulatory component of the ABA receptor; PP2Cs, Type 2C protein phosphatases; SnRK2s, Sucrose non-fermenting-1-related protein kinases 2; ORFs, Open reading frames; *EcPYL*, *Eleusine coracana* Pyrabactin Resistance 1-like; CRE, *Cis*-regulatory elements; ABRE, Abscisic acid responsive element; MeJA, Methyl jasmonate; GARE, Gibberellic acid-responsive elements; ARE, Auxin-responsive elements; MYB, Myeloblastosis viral oncogene homolog; MBS, MYB binding site; NF-Y, Nuclear Factor-Y; TF, Transcription Factor; MCODE, Molecular Complex Detection; MAPK, Mitogen-activated protein kinase; psRNATarget, Plant Small RNA Target; RMSD, Root Mean Square Deviation; NRES, Number of Overlapped Residue; PDB, Protein Data Bank.
