# Supplementary material for: Elucidating the role of pyrabactin-like receptors of finger millet under drought and salinity stress: an insight into in silico, machine learning and molecular approaches
Source: Front Genet. 2025 May 29;16:1598523. doi: 10.3389/fgene.2025.1598523 (PMC12159037; doi:10.3389/fgene.2025.1598523)
Supplement: Supplementary file 3 [file Table1.docx]

**Supp. Table 1:** Retrieved PYL protein sequence (27) after BLASTP search against phytozome database in Finger millet genome

| **Transcript Name** | **Gene Start (bp)** | **Gene End (bp)** | **Chromosome Name** | **Strand** | **PFAM ID** | **PFAM Description** |
| --- | --- | --- | --- | --- | --- | --- |
| ELECO.r07.2AG0108140.1 | 6745596 | 6748419 | 2A | 1 | PF10604 | Polyketide_cyc2 |
| ELECO.r07.2AG0117770.1 | 16588979 | 16589524 | 2A | 1 | PF10604 | Polyketide_cyc2 |
| ELECO.r07.2AG0106820.1 | 5737836 | 5738444 | 2A | -1 | PF10604 | Polyketide_cyc2 |
| ELECO.r07.3BG0283110.1 | 53880214 | 53880825 | 3B | -1 | PF00407 | Bet_v_1 |
| ELECO.r07.3BG0283350.1 | 54043193 | 54043861 | 3B | 1 | PF10604 | Polyketide_cyc2 |
| ELECO.r07.5BG0417310.1 | 5193046 | 5193648 | 5B | 1 | PF10604 | Polyketide_cyc2 |
| ELECO.r07.5BG0450090.1 | 74809940 | 74811451 | 5B | -1 | PF10604 | Polyketide_cyc2 |
| ELECO.r07.5BG0427250.1 | 14229305 | 14229745 | 5B | -1 | PF10604 | Polyketide_cyc2 |
| ELECO.r07.2BG0171370.1 | 14790778 | 14791404 | 2B | 1 | PF10604 | Polyketide_cyc2 |
| ELECO.r07.2BG0160340.1 | 5566370 | 5566972 | 2B | -1 | PF10604 | Polyketide_cyc2 |
| ELECO.r07.2BG0161780.1 | 6714154 | 6716983 | 2B | 1 | PF10604 | Polyketide_cyc2 |
| ELECO.r07.1AG0038470.1 | 51299793 | 51300389 | 1A | 1 | PF10604 | Polyketide_cyc2 |
| ELECO.r07.4BG0345520.1 | 10800457 | 10801016 | 4B | -1 | PF10604 | Polyketide_cyc2 |
| ELECO.r07.4BG0340790.1 | 7679871 | 7680320 | 4B | -1 | PF00407 | Bet_v_1 |
| ELECO.r07.4BG0345510.1 | 10798371 | 10799015 | 4B | -1 | PF10604 | Polyketide_cyc2 |
| ELECO.r07.7AG0582720.1 | 48364558 | 48365109 | 7A | 1 | PF10604 | Polyketide_cyc2 |
| ELECO.r07.6BG0477250.1 | 44444683 | 44445266 | 6B | -1 | PF00407 | Bet_v_1 |
| ELECO.r07.3AG0245550.1 | 45627774 | 45628500 | 3A | 1 | PF00407 | Bet_v_1 |
| ELECO.r07.3AG0244970.1 | 45175801 | 45176412 | 3A | 1 | PF00407 | Bet_v_1 |
| ELECO.r07.3AG0244730.1 | 45026141 | 45026809 | 3A | -1 | PF10604 | Polyketide_cyc2 |
| ELECO.r07.5AG0369710.1 | 6215159 | 6215770 | 5A | 1 | PF10604 | Polyketide_cyc2 |
| ELECO.r07.5AG0380140.1 | 16434114 | 16434683 | 5A | -1 | PF10604 | Polyketide_cyc2 |
| ELECO.r07.5AG0401550.1 | 59625111 | 59626595 | 5A | -1 | PF10604 | Polyketide_cyc2 |
| ELECO.r07.1BG0088430.1 | 66773446 | 66774072 | 1B | -1 | PF10604 | Polyketide_cyc2 |
| ELECO.r07.4AG0314360.1 | 11137006 | 11137574 | 4A | -1 | PF10604 | Polyketide_cyc2 |
| ELECO.r07.4AG0314350.1 | 11133576 | 11134126 | 4A | -1 | PF00407 | Bet_v_1 |
| ELECO.r07.4AG0327710.1 | 39477642 | 39478231 | 4A | -1 | PF00407 | Bet_v_1 |
