# Supplementary material for: Elucidating the role of pyrabactin-like receptors of finger millet under drought and salinity stress: an insight into in silico, machine learning and molecular approaches
Source: Front Genet. 2025 May 29;16:1598523. doi: 10.3389/fgene.2025.1598523 (PMC12159037; doi:10.3389/fgene.2025.1598523)
Supplement: Supplementary file 4 [file DataSheet3.pdf]

PROCHECK

Ramachandran Plot

saves

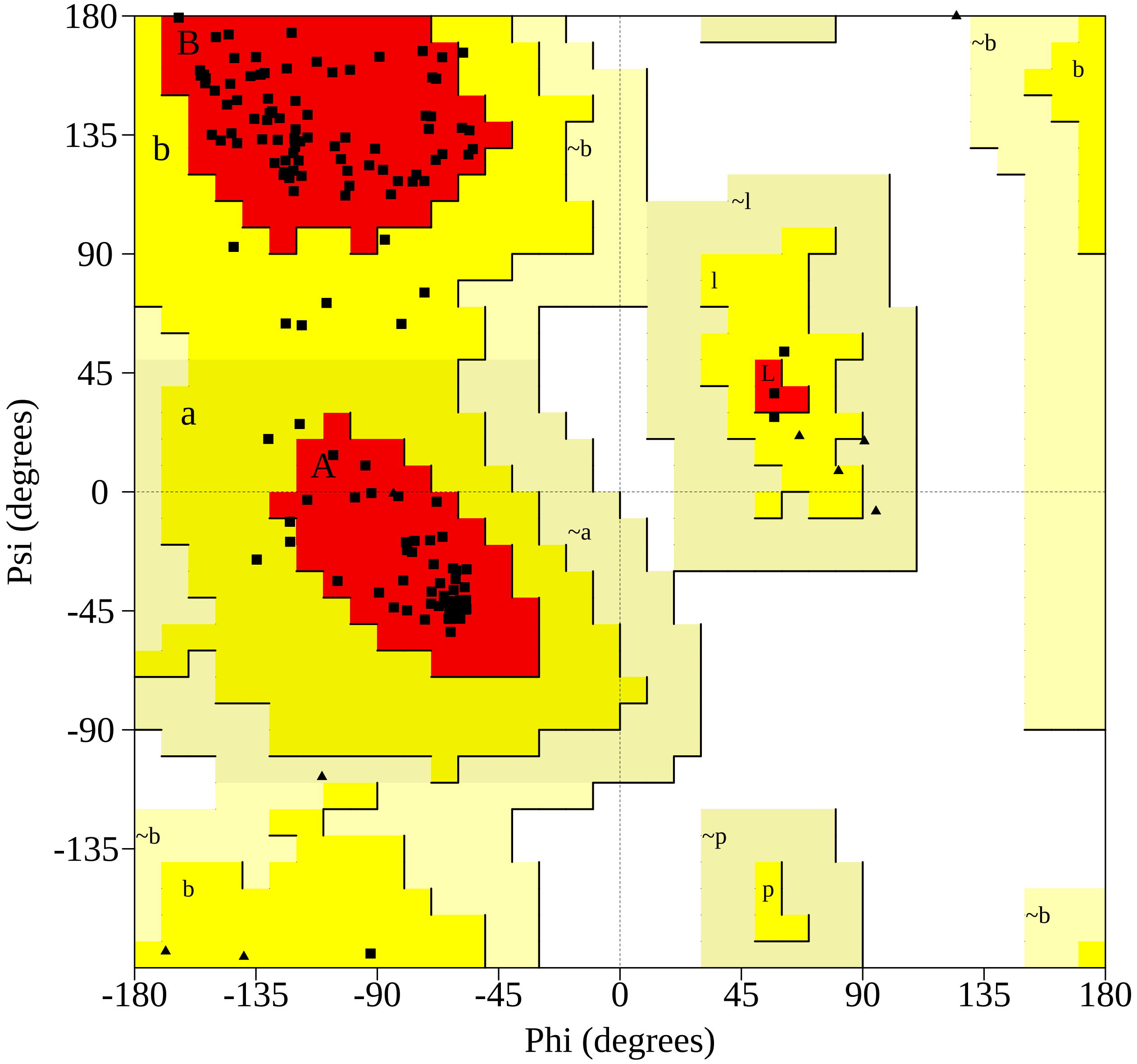

Plot statistics

Residues in most favoured regions [A,B,L]	128	88.9%
Residues in additional allowed regions [a,b,l,p]	16	11.1%
Residues in generously allowed regions [~a,~b,~l,~p]	0	0.0%
Residues in disallowed regions	0	0.0%
	----	------
Number of non-glycine and non-proline residues	144	100.0%
Number of end-residues (excl. Gly and Pro)	2	
Number of glycine residues (shown as triangles)	9	
Number of proline residues	8	

Total number of residues	163	

Based on an analysis of 118 structures of resolution of at least 2.0 Angstroms and R-factor no greater than 20%, a good quality model would be expected to have over 90% in the most favoured regions.

saves_01.ps
