# Supplementary material for: Elucidating the role of pyrabactin-like receptors of finger millet under drought and salinity stress: an insight into in silico, machine learning and molecular approaches
Source: Front Genet. 2025 May 29;16:1598523. doi: 10.3389/fgene.2025.1598523 (PMC12159037; doi:10.3389/fgene.2025.1598523)
Supplement: Supplementary file 5 [file Table2.docx]

Supp. Table 2. Cis regulatory elements in promoter region of EcPYLs genes

| fn | **Cis regulatory elements (CRE)** | **motif** | **function** | **EcPYL1-1A** | **EcPYL2-1B** | **EcPYL3-2A** | **EcPYL4-2A** | **EcPYL5-2A** | **EcPYL6-2B** | **EcPYL7-2B** | **EcPYL8-2B** | **EcPYL9-3A** | **EcPYL10-3B** | **EcPYL11-5A** | **EcPYL12-5A** | **EcPYL13-5B** | **EcPYL14-5B** |
| --- | --- | --- | --- | --- | --- | --- | --- | --- | --- | --- | --- | --- | --- | --- | --- | --- | --- |
| **Hormone responsive** | ABRE | CGTACGTGCA/ ACGTG | cis-acting element involved in the abscisic acid responsiveness | 4 | 4 | 2 | 4 | 0 | 1 | 2 | 0 | 5 | 4 | 2 | 2 | 3 | 1 |
|  | TGA-element | AACGAC | auxin-responsive element | 3 | 3 | 0 | 0 | 0 | 0 | 0 | 0 | 0 | 0 | 0 | 0 | 0 | 0 |
|  | TGACG/ CGTCA-motif | TGACG / CGTCA | cis-acting regulatory element involved in the MeJA-responsiveness | 5 | 4 | 5 | 0 | 6 | 4 | 0 | 5 | 2 | 2 | 0 | 6 | 1 | 6 |
|  | P-box | CCTTTTG | gibberellin-responsive element | 0 | 0 | 0 | 0 | 2 | 0 | 0 | 0 | 0 | 0 | 0 | 0 | 0 | 0 |
|  | GARE-motif | TCTGTTG | gibberellin-responsive element | 0 | 0 | 0 | 0 | 0 | 0 | 0 | 1 | 0 | 0 | 0 | 0 | 0 | 0 |
|  | TCA-element | TCAGAAGAGG | cis-acting element involved in salicylic acid responsiveness | 0 | 0 | 0 | 0 | 0 | 0 | 0 | 1 | 0 | 0 | 0 | 0 | 0 | 0 |
|  | AuxRR-core | GGTCCAT | cis-acting regulatory element involved in auxin responsiveness | 0 | 0 | 0 | 0 | 0 | 0 | 0 | 0 | 0 | 0 | 0 | 1 | 0 | 1 |
| **Light responsive** | G-box | CACGTC | cis-acting regulatory element involved in light responsiveness | 1 | 1 | 1 | 4 | 0 | 2 | 1 | 0 | 2 | 2 | 3 | 1 | 3 | 1 |
|  | GATA motif | AAGATAAGATT | part of a light responsive element | 0 | 0 | 2 | 0 | 1 | 2 | 0 | 0 | 1 | 1 | 0 | 1 | 1 | 0 |
|  | Box 4 | ATTAAT | part of a conserved DNA module involved in light responsiveness | 1 | 1 | 0 | 1 | 1 | 0 | 1 | 1 | 0 | 1 | 1 | 0 | 1 | 0 |
|  | ACE | GCGACGTACC | cis-acting element involved in light responsiveness | 0 | 0 | 0 | 0 | 1 | 0 | 0 | 1 | 1 | 0 | 0 | 0 | 0 | 0 |
|  | C-box | ACGAGCACCGCC | cis-acting element involved in light responsiveness | 0 | 0 | 0 | 0 | 1 | 0 | 0 | 0 | 1 | 1 | 1 | 1 | 1 |  |
|  | MRE | AACCTAA | MYB binding site involved in light responsiveness | 0 | 0 | 0 | 0 | 1 | 0 | 0 | 0 | 0 | 0 | 0 | 0 | 0 | 0 |
|  | Sp1 | GGGCGG | light responsive element | 0 | 0 | 1 | 0 | 0 | 1 | 0 | 0 | 1 | 0 | 2 | 2 | 2 | 2 |
| **Stress responsive** | TC-rich repeats | ATTCTCTAAC | cis-acting element involved in defense and stress responsiveness | 1 | 1 | 1 | 0 | 0 | 0 | 0 | 0 | 0 | 0 | 0 | 0 | 0 | 0 |
|  | LTR | CCGAAA | cis-acting element involved in low-temperature responsiveness | 0 | 0 | 0 | 2 | 0 | 0 | 1 | 0 | 1 | 1 | 0 | 0 | 0 | 0 |
|  | MBS | CAACTG | MYB binding site involved in drought-inducibility | 0 | 0 | 1 | 1 | 0 | 1 | 1 | 0 | 0 | 0 | 3 | 0 | 3 | 0 |
| **Growth and Development responsive** | RY-element | CATGCATG | cis-acting regulatory element involved in seed-specific regulation | 1 | 1 | 2 | 0 | 0 | 1 | 0 | 0 | 0 | 0 | 0 | 0 | 0 | 0 |
|  | circadian | CAAAGATATC | cis-acting regulatory element involved in circadian control | 0 | 0 | 0 | 1 | 0 | 0 | 1 | 0 | 0 | 0 | 0 | 0 | 0 | 0 |
| **Transcription responsive** | CAAT-box | CCAAT | common cis-acting element in promoter and enhancer regions / involved in flowering regulation | 4 | 3 | 4 | 5 | 9 | 4 | 6 | 6 | 4 | 5 | 2 | 5 | 2 | 3 |
|  | TATA-box | TATATA / TATA | core promoter element around -30 of transcription start | 2 | 2 | 9 | 16 | 18 | 7 | 12 | 15 | 12 | 13 | 4 | 13 | 2 | 12 |
