# Supplementary material for: Elucidating the role of pyrabactin-like receptors of finger millet under drought and salinity stress: an insight into in silico, machine learning and molecular approaches
Source: Front Genet. 2025 May 29;16:1598523. doi: 10.3389/fgene.2025.1598523 (PMC12159037; doi:10.3389/fgene.2025.1598523)
Supplement: Supplementary file 6 [file DataSheet1.pdf]

PROCHECK

# Ramachandran Plot

## saves

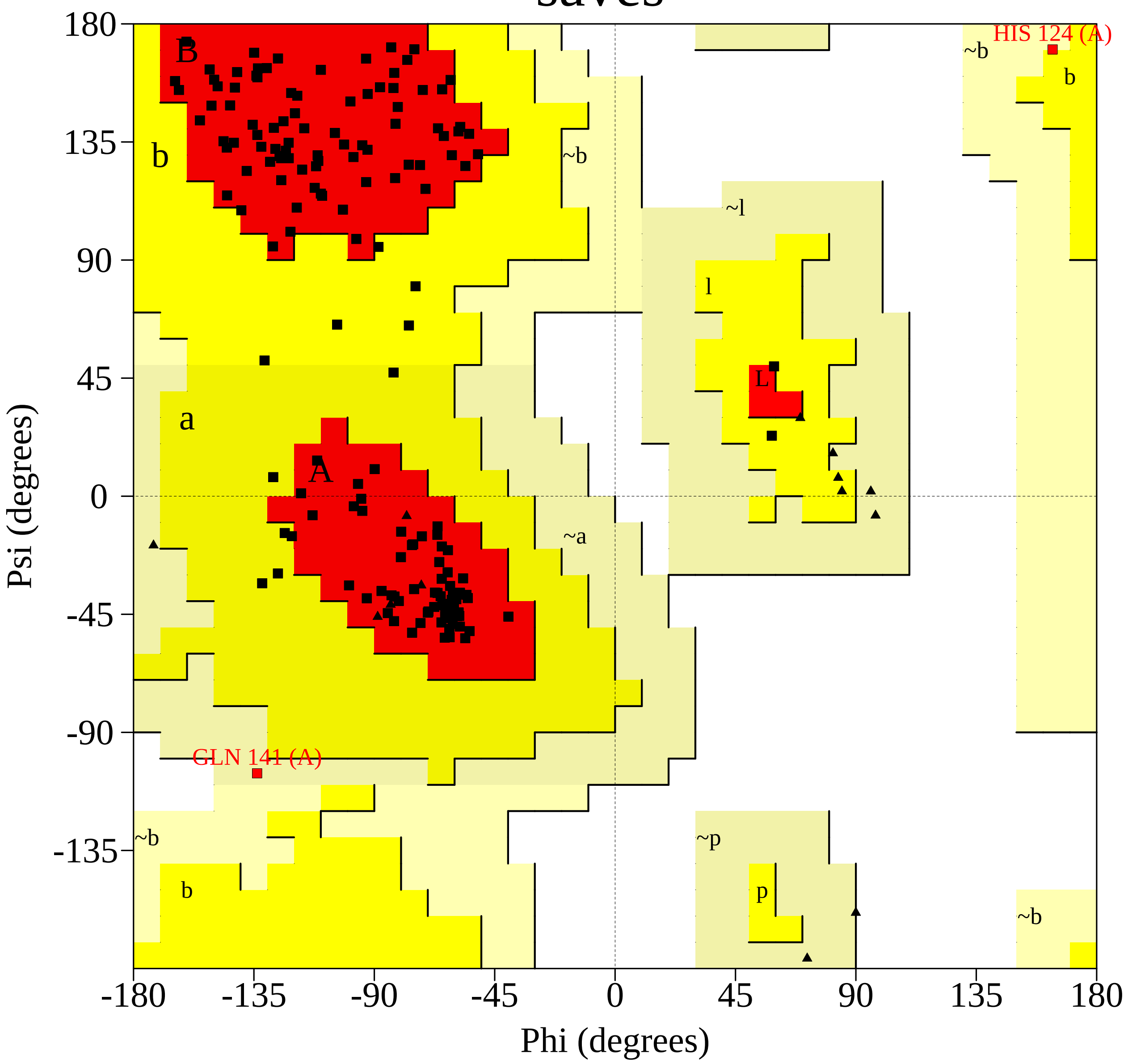


Plot statistics

| | | |
|---|---|---|
| Residues in most favoured regions [A,B,L] | 150 | 92.0% |
| Residues in additional allowed regions [a,b,l,p] | 11 | 6.7% |
| Residues in generously allowed regions [~a,~b,~l,~p] | 2 | 1.2% |
| Residues in disallowed regions | 0 | 0.0% |
| | ---- | ------ |
| Number of non-glycine and non-proline residues | 163 | 100.0% |
| Number of end-residues (excl. Gly and Pro) | 1 | |
| Number of glycine residues (shown as triangles) | 13 | |
| Number of proline residues | 9 | |
| | ---- | |
| Total number of residues | 186 | |

Based on an analysis of 118 structures of resolution of at least 2.0 Angstroms and R-factor no greater than 20%, a good quality model would be expected to have over 90% in the most favoured regions.

saves_01.ps
