# Supplementary material for: Elucidating the role of pyrabactin-like receptors of finger millet under drought and salinity stress: an insight into in silico, machine learning and molecular approaches
Source: Front Genet. 2025 May 29;16:1598523. doi: 10.3389/fgene.2025.1598523 (PMC12159037; doi:10.3389/fgene.2025.1598523)
Supplement: Supplementary file 7 [file Table3.docx]

Supp Table 3 Putative miRNA and their targeted PYLs genes in finger millet.

| miRNA_Acc. | Target_Acc. | e-value | UPE (Unpaired Energy) | Target_start | Target_end | miRNA_aligned_fragment | Target_aligned_fragment | Inhibition |
| --- | --- | --- | --- | --- | --- | --- | --- | --- |
| Eco-miRN5585 | EcPYL10-3B | 4.5 | -1 | 248 | 267 | AACGGGCUGCACUGCUGGCC | CGGCGGCGGUGUGGUCCGUG | Cleavage |
| Eco-miRN5585 | EcPYL9-3A | 4.5 | -1 | 248 | 267 | AACGGGCUGCACUGCUGGCC | CGGCGGCGGUGUGGUCCGUG | Cleavage |
| Eco-miR529a | EcPYL4-2A | 5 | -1 | 338 | 358 | GCUGUACCCUCUCUCUUCUUC | GAAGCACAGAGAGGUUAGAGC | Cleavage |
| Eco-miRN34a | EcPYL3-2A | 5 | -1 | 126 | 145 | AGUCCUCGUGCUGCAUCCCU | GGUGACGCAGCGCGUGGACG | Cleavage |
| Eco-miRN34a | EcPYL6-2B | 5 | -1 | 126 | 145 | AGUCCUCGUGCUGCAUCCCU | GGUGACGCAGCGCGUGGACG | Cleavage |
| Eco-miRN5585 | EcPYL9-3A | 5 | -1 | 218 | 237 | AACGGGCUGCACUGCUGGCC | CGGCGGUGGUGCAGCACGUG | Cleavage |
